# The local government bond channel of China's monetary policy

**DOI:** 10.1371/journal.pone.0354727

**Published:** 2026-08-18

**Authors:** Jinzhi Bai, Hanlin Xia, Lin Zhang

**Affiliations:** Institute of Chinese Financial Studies, Southwestern University of Finance and Economics, Chengdu, Sichuan, China; University of Salerno: Universita degli Studi di Salerno, ITALY

## Abstract

China's economic development and change are significantly influenced by local governments. By affecting the cost of borrowing for local governments, monetary policy can have an impact on their fiscal policies and, eventually, the actual economy. After providing empirical evidence to support the existence of the local government bond channel of monetary policy in China, this paper builds a DSGE model with a multi-region and multi-level government structure in order to quantitatively analyze the impact of the local government bond channel of monetary policy. The findings indicate that local government borrowing costs have a major impact on the transmission of monetary policy. The stimulus impact of monetary policy is significantly increased when local governments may borrow at the risk-free rate, resulting in an output response that is more than twice as large as that of the baseline model. With some cross-regional financial spillovers among local governments, the output response at the average response is lower than in the benchmark model, but the output response at the 75th percentile bond rate is more than twice as large. Since local government borrowing costs respond differently to monetary policy, there are notable geographical consequences of monetary policy. Competition among local governments reduces the impact of monetary policy transmission. This paper's quantitative analysis offers a fresh analytical viewpoint on how China's monetary policy is transmitted during periods of transition.

## Introduction

**Fig 1:**
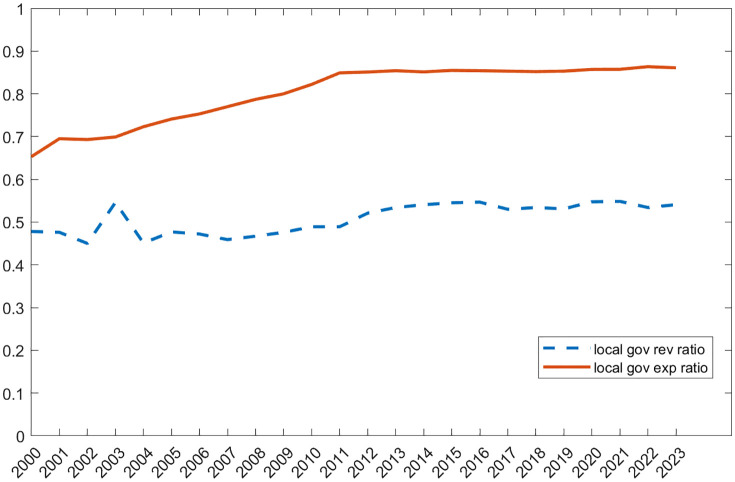
The proportion of local government expenditure and income since 2000. **Local gov exp ratio** indicates the proportion of local governments’ expenditures to total expenditures (sum of central and local government expenditures), and **local gov rev ratio** indicates the proportion of local governments’ revenues to total revenues (sum of central and local government revenues).Our data comes from the National Bureau of Statistics of China.

The market for local government bonds has grown quickly due to the funding demands of local governments, and the size of these bonds is increasing. The pressure to pay back its debt is growing, with the remaining chengtou bonds (a kind of implicit local government bond) totaling 15.52 trillion yuan and the remaining local government debt totaling 47.53 trillion yuan in 2024. The Ministry of Finance reports that interest payments on local government bonds would total 1.35 trillion yuan in 2024, and that the interest payment costs will account for about 4.89% of the local comprehensive financial resources, up from 3% in 2019.Since reform and opening up, China has had a unique institutional structure with a high degree of political authority centralization and a high degree of economic system decentralization [1]. Local governments have been granted significant autonomy to make decisions regarding regional operations since china’s tax-sharing system was reformed in 1994. They are the monopolizers of scarce resources, the creators and overseers of regional regulations and rules, the providers of public goods, and the advocates of economic development within their jurisdictions [2]. Furthermore, it gradually broke the absolute dominance of the central government over financial resources, and the dominant power of financial resources was delegated to local governments. Thus, the primary executor of fiscal income and spending choices in the framework of the Chinese fiscal decentralization system is the local government [3]. However, as seen in the Fig 1, local governments suffer from a serious vertical fiscal imbalance, meaning that a high ratio of local expenditures to total expenditures and a low ratio of local revenues to total revenues. The current gap in local fiscal imbalances is mainly supported by central transfers and local government bond issuance (including chengtou bonds).

**Fig 2:**
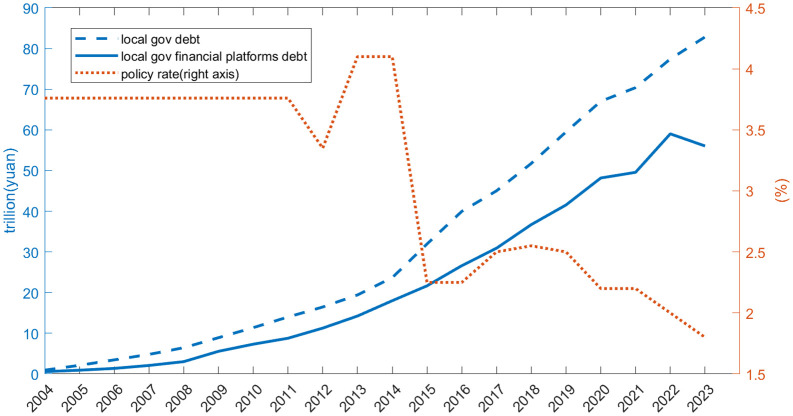
Policy rates and the size of local government debt. **Local gov debt** denotes the size of local government debt is calculated by the method of Liu and Li (2021) [4], **local gov financial platforms debt** denotes the size of local government debt is calculated by the method of Cao et al.(2019) [5], and policy rate indicates the 7-day reverse repo operation rate. Relevant data are from Wind, China Financial Statistics Yearbook and China Fixed Assets Statistics Yearbook.

Local government bonds have an important impact on the transmission of monetary policy. With the primary market issuance rate and secondary market transaction rate of bonds directly influencing the average financing cost of non-financial enterprises, the central bank monetary policy regulatory framework in China is undergoing a transition from a quantity-based to a price-based approach. The transmission of monetary policy through the bond market is also playing an increasingly evident “signaling” role. As a key conduit for the central bank's open market operations instruments, bonds are becoming more and more significant in the process by which monetary policy is transmitted. As shown in Fig 2, during the sample period, policy interest rates generally trended downward, while the scale of local government debt continued to expand. The temporal correlation between these two variables underscores the importance of examining whether and how monetary policy influences the dynamics of local government debt. Combined with the fact that local government spending in China continues to rise, this paper posits that a monetary policy channel for local government debt may exist in China—that is, monetary policy can influence local fiscal policy (local government debt and investment), thereby affecting local economic development. This paper will conduct a more rigorous analysis and verification of this hypothesis in the subsequent sections on empirical analysis and theoretical modeling.

This paper starts with regression analysis and an instrumental variable vector autoregression model (Proxy SVAR) to empirically demonstrate the existence of the local government bond channel of monetary policy in China. Regression analysis results indicate that there is a local government bond channel for monetary policy transmission through which monetary policy shocks influence local fiscal policy through borrowing costs. This transmission is cross-regionally heterogeneous, and competition among local governments significantly inhibits this transmission. The presence of a local government bond channel for the transmission of monetary policy is also examined via the Proxy SVAR model.

Based on the empirical analysis, this paper develops a theoretical model for simulation analysis. In addition to incorporating the local government bond channel of monetary policy, the model incorporates the following facts characterizing the China's economy: (1) China has several levels of government, and the fiscal policies of both the national and local governments have an impact on the economy simultaneously; (2) local governments primarily depend on investment to drive the growth of the local economy and optimize their behaviors based on intrinsic incentives;(3) Local governments compete with one another, which leads to competition for debt; and (4) Local financial frictions cause local governments’ borrowing costs to diverge from the risk-free rate, with excessive spreads and geographical heterogeneity. In order to quantitatively analyze the size of the local government bond channel of monetary policy and the effect of financial frictions on the local government's fiscal response to macroeconomic shocks, this paper builds a two-region dynamic stochastic general equilibrium model containing the central government, local governments, financial institutions, households, and firms.

This paper is relevant to the literature on the following areas: the study of the bond market transmission of monetary policy, the study of the regional effects of monetary policy, and the study of fiscal and debt problems under a multi-tiered government. First, this paper complements studies by Gilchrist et al. (2015), Sensarma and Bhattacharyya (2016), Burger et al. (2017), Szczerbowicz (2018), Fang et al. (2023), El-Shagi and Jiang (2023), which examine the impact of conventional and unconventional monetary policies on the treasury bond market or corporate bond market [6–11], while this paper emphasizes the impact of monetary policy on the local government bond market. At the same time, this paper combines monetary union and bond market transmission of monetary policy, complementing the literature on fiscal and monetary policy in monetary unions, such as Beetsma and Jensen(2005), Nakamura and Steinsson(2014), Farhi and Weining(2017) [12–14].

The most relevant work for this paper is Wilson (2025) [15], which examines the municipal bond channel of monetary policy in the US, while this paper emphasizes the local government bond channel of monetary policy in China, which differs from it in two main ways: First, the state and local governments in the United States are very different from those in China in terms of their functions, with China’s local governments emphasizing more on their function of economic development, and not just the provision of public goods. Second, local governments in China face fierce competition among themselves, which can have a greater impact on the transmission effect of monetary policy. These two points make the modeling framework for the local government bond channel of monetary policy different from Wilson (2025).

In addition, through a local government model of a monetary union, this paper complements studies related to the regional effects of monetary policy, such as Carlino and Defina (1998, 1999), Owyang and Wall (2003), Fielding and Shields (2011), Adam Elbourne and Jakob de Haan (2009), Huang and Qin (2017), Tang (2023), Tsang (2024) [16–23]. Regarding the reasons for the regional effects of monetary policy, this paper analyzes the local government borrowing cost perspective and argues that the heterogeneity of local government borrowing cost responses leads to regional differences in the transmission of monetary policy, which is different from the existing literature, such as the credit channel and the interest rate channel [24,25], regional trade and capital mobility [26], regional productivity level differences [27], real estate market [28], regional competition [29], financial development level and local government land finance dependence [30].

Finally, in terms of research on fiscal policy issues under multilevel government, Zhu and Xu (2018) constructed a multilevel government model with the central government and two regional governments to analyze the dynamic impact of local government fiscal policy on China's macroeconomy under fiscal decentralization [31]; Wang et al. (2020) used a two-region model to study the role of fiscal decentralization on the structural imbalance of the economy and its intrinsic mechanism [32]; Xiong et al. (2022) analyze the regional spillover effects of local government hidden debt by constructing a dynamic general equilibrium model that includes local governments and the central government in two regions [33]. This paper has in common with the above studies the presence of multi-regional and multi-level governments, but this paper's main goal is to examine how monetary policy affects local fiscal policy and quantify the influence of financial frictions on the transmission of monetary policy.

Compared to the existing literature, contributions of this paper include the following:(1) Confirming the existence of the local government bond channel of monetary policy in China enriches the relevant studies on monetary policy transmission based on the Chinese context. The local government bond channel of monetary policy provides additional economic stimulus beyond the traditional channel. (2) A new way for monetary policy to affect local government fiscal policy is by include the local government bond channel of monetary policy in the theoretical model to investigate how local competition affects the transmission of monetary policy. (3) Based on the perspective of local governments’ borrowing costs, there is significant heterogeneity in the transmission of monetary policy across regions, complementing the study of regional effects of monetary policy.

The remainder of the paper is organized as follows. The Empirical Analysis section confirms the existence of the local government bond channel of monetary policy in China. In Theoretical Models section, we construct a multi-level and multi-regional DSGE model. In Quantitative Analysis section, we calibrate the model and quantitatively analyze the local government bond channel of China's monetary policy. Finally, the Conclusions and Implications section summarizes the conclusions and proposes policy implications.

## Empirical analysis

With the current huge scale of local government debt, its debt pressure will be affected by monetary policy. Monetary policy affects local government borrowing costs, which in turn has a significant impact on local government fiscal policy and incentives. Next, this paper will empirically analyze the existence of the local government bond channel of monetary policy through local government borrowing costs which in turn affects local government fiscal policy and ultimately the real economy.

This section is divided into five parts: first, a basic statistical analysis of the local government bond market and related yields; second, a strategy for identifying monetary policy shocks; third, an analysis of the response of local government bond yields to monetary policy shocks, and emphasizes the heterogeneity of the response among local governments as well as the impact of local government competition; fourth, the impact of local government bond yields on local government fiscal policy; and fifth, proxy SVAR of analysis the monetary policy shock.

### Local government bond market

At the end of the twentieth century, relevant laws and regulations clearly stipulated that local governments and their departments should not act as borrowers or guarantors, but did not explicitly prohibit the indirect raising of debt through financing platform companies, which provides local governments with the possibility of raising debt through the establishment of financing platform companies. With the sudden outbreak of the Asian Financial Crisis in 1997, the central government issued special treasury bonds and then transferred them to the local governments to meet the demand for funds for the local economic construction. At the same time, in order to circumvent the limitations on debt raising under the Budget Law, local governments have established financing platform companies, whose main financing methods are bank loans and bond issuance. As the industrialization and urbanization process progresses, the number of financing platform companies has significantly increased, and their business scope and financing methods tend to be diversified, such as obtaining policy loans in the form of“bank-government cooperation”and “bundled loans”, and using the fiscal budget or special funds and revenues from governmental funds as sources of debt servicing. In order to cope with the international financial crisis in 2008, the central government launched the“4 trillion yuan”investment plan(of which local governments were responsible for 2.82 trillion yuan), and the financing platform companies were further developed and gradually became an important investment and financing tool for local governments. At present, financing platform companies are facing a market-oriented transformation, changing their role from “local government investment and financing entities” to “urban infrastructure operators” or state-owned enterprises engaged in other business operations (housing construction and ecological environment improvement, etc.).

In addition to through financing platform companies, local government bonds have become the most important source for local governments to meet their financing needs. The “Measures for Budget Management of Local Government Bonds in 2009,” which were released by the Ministry of Finance (MOF) on February 18, 2009, permit some local governments to borrow debt directly. According to this document, the Ministry of Finance began to issue government bonds on behalf of local governments (at the provincial level) and included them in local governments fiscal budgets, and constituting local governments debt; Before the maturity of the bond, the local government needs to pay the principal and interest repayment funds into the central financial account, and the Ministry of Finance will handle the principal and interest repayment business on behalf of the local government, which is known as the“issuance and repayment on behalf of the local government bonds”mode. During the 2009–2011 period, local governments maintained an annual bond issuance scale of 200 billion yuan. Using a mechanism known as “self-issuance and repayment,” the central government permitted Shanghai, Zhejiang, Guangdong, and Shenzhen to issue bonds on a trial basis within the allowed quota in 2011. The Ministry of Finance would then handle the debt payments on their behalf. The addition of Jiangsu and Shandong as pilot regions for “self-issuance and self-repayment” of local government bonds was authorized by the central government in 2013. Beijing, Qingdao, Jiangxi, and Ningxia have joined the six pilot provinces to implement the “2014 Local Government Bonds Self-Issuance and Self-Repayment Pilot Measures,” which was promulgated by the Ministry of Finance on May 22, 2014. This means that the localities will issue the bonds on their own and manage the repayment of the principal and interest on their own. The amount of local government bonds issued rose to 250 billion yuan in 2012, 350 billion yuan in 2013, and 400 billion yuan in 2014. The legal restrictions on local governments issuing bonds were lifted by January 1, 2015, when the new Budget Law made it clear that local governments must raise debt through self-issuing and self-repaying government bonds under budgetary constraints [34]. As a result, 2015 is seen as the beginning of China's local government bond market's modernization and standardization. Since 2015, the scale of its local government bonds has been gradually increasing, and it has become the main way for local governments to raise funds. At present, local government debt mainly consists of local government bonds within the budget as well as off-budget hidden debt (mainly financing platform company bonds, or called chengtou bonds). As financing platform companies are largely set up to carry out local government-related functions, financing platform company bonds are regarded here as a special kind of local government bonds.

Since China's local government bond market underwent a significant institutional transformation in 2015, the benchmark sample of local government bonds starts that year. Following the implementation of the revised Budget Law, local governments became responsible for self-issuance and self-repayment of bonds under explicit budget constraints. Before 2015, the Ministry of Finance's proxy issue and local government finance vehicles were the primary sources of borrowing for local governments. This suggests that administrative arrangements, rather than market pricing, had a significant influence on observed bond yields. Consequently, employing official local government bonds issued prior to 2015 would decrease the identification of the monetary transmission mechanism and induce structural cracks. Starting the sample in 2015 allows us to analyze a relatively homogeneous institutional regime in which local government bond yields reflect market-based financing costs.

Furthermore, the methodologies used in this article to determine the yield to maturity and credit spreads for local government bonds and chengtou bonds, as well as the choice of maturities, are explained in depth below. The yield to maturity of treasury bonds is calculated with reference to ang et al. (2023) [35], specifically, the yield curve of treasury bonds is first estimated on a daily basis using the daily closing price of treasury bonds, and then the curve is used to calculate the yield to maturity of virtual treasury bonds with the same maturity and coupon rate as the corresponding chengtou bonds in order to adjust for the impact on yield to maturity due to the difference in the cash flow structure of the municipal bonds and the treasury bonds. The credit spread of chengtou bonds is the difference between the yield to maturity of chengtou bonds and the yield to maturity of the corresponding virtual treasury bonds. The credit spread of local government bonds is calculated in a similar way. After calculating the yield to maturity and credit spread for each individual bond, the weighted average yield to maturity and credit spread are obtained using a balance-weighted average, calculated as follows:


$$\begin{array}{c} \text{the weighted average credit spread }=\sum \left(\frac{\text{balance of the individual bond}}{\text{total balance of individual bond}}*\text{spread of the individual bond}\right) \end{array}$$

This indicator more accurately captures the real financing expenses local governments face because newly issued bonds have different maturities. The balance-weighted average offers a more realistic view of the whole cost of debt financing that is pertinent to fiscal decision-making than metrics that concentrate on a single maturity.

By the December 2024, the balance of local government debt was 47.54 trillion yuan, and the balance of chengtou bonds was 15.52 trillion yuan. Local governments repaid 2.99 trillion yuan in principal and 1.35 trillion yuan in interest in 2024. Due to the differences in endowment and level of economic development among local governments, there are large differences in debt size and debt ratios among local governments. In addition to the differences in debt size, there are also significant differences in local government bond yields, and Table 1 gives basic statistics on the yields of local government bonds and chengtou bonds. Heterogeneity exists among different local governments both from the perspective of yield to maturity and credit spreads. It should be emphasized that there is more significant heterogeneity among chengtou bonds of different local governments. Table 1 shows that the standard deviation of the yield to maturity of local bonds is 0.48, while the standard deviation of the yield to maturity of municipal bonds is 0.86, the standard deviation of the credit spreads of local government bonds is 0.14, and the standard deviation of the credit spreads of chengtou bonds is 0.48, which reflect the existence of the heterogeneity of the cost of borrowing among the local governments, which provide a realistic basis for the theoretical modeling analysis in the subsequent section.

**Table 1:** Basic statistics on local government bond yields and credit spreads.

	mean	standard deviation	25th	50th	75th
LGB yield	3.23	0.48	2.82	3.21	3.54
LGB credit spreads	0.30	0.14	0.21	0.27	0.37
CB yields	4.37	0.86	3.62	4.28	5.05
CB credit spreads	1.29	0.48	0.84	1.28	1.69

Note: Data from Wind. LGB stands for local government bonds, and CB stands for chengtou bonds. The sample period for local government bonds is from 2015 to 2023. The sample period for chengtou bonds is from 2009 to 2023. Both credit spreads and yields to maturity are calculated as balance-weighted averages.

### Identification strategies for monetary policy shocks

In this paper, we identify monetary policy shocks through trading data from financial markets, which is called the high-frequency identification method. Compared to traditional monetary policy identification methods (e.g., vector autoregression methods), the high-frequency identification method can make sure the exogeneity of the monetary policy shocks, making it more reliable when analyzing complex problems (especially financial problems). High-frequency identification methods use daily or intraday trading data from financial markets to identify exogenous shocks to monetary policy [36], which can introduce more information from financial market trading data to better address causal identification challenges [37].

The basic idea of the high-frequency identification method is as follows: under the assumption of the complete information condition, the macro fundamentals are regarded as unchanged during a relatively short period of time of the monetary policy adjustment, and therefore, the price changes of the financial market products (e.g., the interbank market interest rate), which are regarded as unaffected by other macro-events, can be attributed to the price changes due to the unanticipated monetary policy shocks. High-frequency identification is specified by using price changes in financial market products during a shorter window (e.g., 30 minutes) within the trading day as price changes due to monetary policy shocks [36].

The high-frequency identification method in this paper is based on the daily price series of 1-year interest rate swaps on the 7-day interbank repo fixing rate (FR007) to construct a series of exogenous monetary policy shocks in China from January 2009 to December 2023. The data source is the Bloomberg database. Since monetary policy adjustments have a direct and timely impact on FR007, and China's 1-year FR007-IRS is highly traded and marketed, this paper selects the 1-year FR007-IRS as the underlying price indicator for China's exogenous monetary policy shock series [38].

Since most of the important monetary policy announcements in China, such as reserve requirement ratio adjustments and benchmark interest rate adjustments, are released during non-trading hours in most financial markets, there is no suitable intraday price window, so this paper selects the day time window to calculate the price changes in financial markets. In this paper, we use the changes in the 1-year FR007 interest rate in the Chinese interest rate swap (IRS) market around the announcement date of the monetary policy adjustment to identify monetary policy shocks in China. The specific calculation rules are: if the policy announcement occurs when the financial market opens and on a business day, then the difference between the closing price on the day of the announcement minus the closing price on the previous business day is calculated; if the policy announcement occurs after the market closes and on a business day, then the difference between the closing price on the next day minus the closing price on the day of the announcement is calculated; and If the policy announcement is made during a weekend or holiday, then the difference between the closing price on the first business day after the announcement minus the closing price on the last business day before the announcement is calculated. Based on this rule, we construct the changes in the 1-year FR007-IRS before and after the policy announcements from 2009 to 2023 and use it as an instrumental variable for monetary policy shocks in China.

In this paper, we define China's monetary policy announcements as the concatenation of the following three types of announcements: announcements by the People's Bank of China (PBOC) announcing adjustments to the legal reserve ratio, announcements announcing adjustments to the benchmark lending rate, and quarterly monetary policy implementation reports. During the sample interval (January 2009 to December 2023), these three types of monetary policy announcements occur 33 times, 13 times and 59 times, respectively. The daily sequence of monetary policy shocks corresponding to the monetary policy announcements constructed in this paper is notated as $m_{t}$, positive for accommodative monetary policy shocks and negative for tightening monetary policy shocks. The correlation coefficients of this monetary policy shock series with the reserve ratio and the benchmark lending rate are −0.67 and −0.75, respectively, indicating that the series extracted by the high-frequency identification method can reflect the direction of monetary policy adjustment.

### The effect of monetary policy shocks on maturity yields and credit spreads

This section estimates the impact of monetary policy shocks on maturity yields and credit spreads of local government and corporate bonds. To do so, we estimate the following regressions:


$$\begin{array}{c} \Delta y_{t}=\alpha +\beta m_{t}+\varepsilon_{t} \end{array}$$ (1)

where $m_{t}\;$is a monetary policy shock, $\Delta y_{t}$ represents the day-to-day change in maturity yields and credit spreads of the underlying assets (local government bonds, municipal bonds, and corporate bonds) around the date of the central bank's monetary policy announcement. The coefficients β denote the percentage point change in the yield to maturity of local government bonds or corporate bonds resulting from each percentage point of monetary policy shock.

Table 2 shows that all the coefficient valuations of yields and credit spreads are significantly positive, i.e., accommodative monetary policy leads to a decrease in the yields to maturity and credit spreads of the relevant bonds. In particular, local government bond maturity yields have the smallest response, while corporate bond maturity yields have the largest response, and the response of municipal bond maturity yields is in the middle, which is consistent with the findings of Kamber and Mohanty (2018) [38]. As for the credit spreads, which reflect the risk of the underlying bond assets, the above coefficients reflect the fact that the accommodative monetary policy reduces the risk of these bonds, which is the same as the relevant studies in the literature. It's noteworthy to see that corporate bonds react to monetary policy shocks far more strongly than municipal and local government bonds, which may indicate that the responses of latter two types of bonds are dampened. For example, the estimate of 0.24 for local government bonds suggests that the yield to maturity of local government bonds would fall by about 0.24 percentage points along with a 1 percentage point cut in the policy rate, while the estimate of 0.46 for corporate bonds suggests that the yield to maturity of corporate bonds would fall by about 0.46 percentage points along with a 1 percentage point cut in the policy rate. The response of corporate bonds is almost twice as large as the response of local government bonds, suggesting that relevant agents tend to overestimate the impact of monetary policy on local government fiscal policy if this dampened response is not taken into account.

**Table 2:** Results of baseline estimation.

	LGB yields	LGB credit spreads	CB yields	CB credit spreads	CPB yields	CPB credit spreads
MP shock	0.24 (0.04)	0.11 (0.06)	0.29 (0.09)	0.13 (0.05)	0.46 (0.13)	0.16 (0.07)
N	2252	2252	3751	3751	3751	3751

Note: **LGB** stands for local government bonds, **CB** stands for chengtou bonds, and **CPB** stands for corporate bonds. **MP shock** stands for monetary policy shock. The data range is from January 2015 to December 2023 for local government bonds; the data range is from January 2009 to December 2023 for chengtou bonds and corporate bonds. The purpose of this sample period selection was to improve the comparability of chengtou bonds and local government bonds by removing structural noise brought on by specific institutional limitations. An observation corresponds to a period of days. Each column corresponds to a time-series regression of the associated bond maturity yields and credit spreads on monetary policy shocks. Standard errors are reported in parentheses. To mitigate concerns regarding sample period selection, we restricted the sample of chengtou bonds to the period 2015–2023, aligning it exactly with the sample period for local government bonds, and re-estimated equation (1). The empirical findings demonstrate that the estimated coefficient of the yield on municipal bonds’ response to monetary policy shocks is consistent with the estimate (0.29) from the entire sample (2009–2023) in both sign and significance, and the magnitude is also quite similar (0.31). This suggests that the fundamental finding—that urban investment bonds are more susceptible to monetary policy shocks—remains constant despite the variations in sample periods.

In order to study the heterogeneity of responses among different provincial governments, using equation (1) and doing a regression on the local government bond yield data of each province, we can get the estimates of provincial responses to monetary policy shocks, summarized in Table 3.

**Table 3:** Results of provincial estimation.

East	MP shock	Central	MP shock	West	MP shock
Beijing	0.43 (0.08)	Shanxi	0.24 (0.12)	Neimenggu	0.23 (0.21)
Tianjin	0.28 (0.19)	Anhui	0.26 (0.16)	Guangxi	0.22 (0.04)
Hebei	0.25 (0.14)	Jiangxi	0.23 (0.18)	Chongqing	0.29 (0.09)
Liaoning	0.32 (0.12)	Henan	0.21 (0.14)	Sichuan	0.30 (0.14)
Shanghai	0.47 (0.14)	Hubei	0.30 (0.18)	Guizhou	0.22 (0.16)
Jiangsu	0.35 (0.17)	Hunan	0.27 (0.15)	Yunnan	0.23 (0.09)
Zhejiang	0.39 (0.09)	Jilin	0.19 (0.05)	Shanxi	0.26 (0.08)
Fujian	0.29 (0.21)	Heilongjiang	0.16 (0.04)	Gansu	0.19 (0.05)
Shandong	0.28 (0.11)			Qinghai	0.23 (0.17)
Guangdong	0.38 (0.15)			Ningxia	0.21 (0.06)
Hainan	0.22 (0.21)			Xinjiang	0.17 (0.08)

Note: Due to data restrictions, Tibet Autonomous Region, Hong Kong Special Administrative Region and Macao Special Administrative Region are excluded. Following Shen et al. (2021) for the criteria of dividing **East**, **Central** and **West** [39]. Shanxi in the central region stands for “山西”, and Shanxi in the west region stands for “陕西”. Standard errors are reported in parentheses.

The response of local government bond maturity yields to monetary shocks exhibits significant cross-spatial heterogeneity, as demonstrated in Table 3, with monetary policy having varying effects on borrowing costs. The most responsive provincial government is Shanghai, where a 1 percentage point reduction in the policy rate is accompanied by a decline in the yield to maturity on local government bonds of approximately 0.47 percentage points, which is nearly three times as much as that of the least responsive provincial government (Heilongjiang Province). In terms of regional differences, local governments in the eastern region generally reacted somewhat more strongly than those in the central and western regions, and within each region, local governments with better economic development reacted relatively more strongly.

The heterogeneity of responses among provincial governments may stem from local financial frictions. In terms of the history of local economic development, much of the development of local economies comes from the advancement of industrialization and urbanization, and local government competition is an important driving force for local economic development, which also affects local governments’ responses to macroeconomic policies. The more competitive local governments are, the relatively fewer financial resources they face. The relatively imperfect system related to the bond market, the greater financial frictions they suffer, and the less bargaining space for borrowing costs local governments face, and thus the smaller the response of local government bond yields and credit spreads.

To explore the impact of local government competition on the response of maturity rates to monetary policy shocks, we employ balanced panel data at the province level. In order to use more information from the data, the following regressions focus on the maturity yields and credit spreads of chengtou bonds:


$$\begin{array}{c} \Delta y_{it}=\alpha +\beta m_{t}+\gamma X_{it}+\delta m_{t}{X_{it}+\varepsilon }_{t} \end{array}$$ (2)

where $\Delta y_{it}$ denotes the change in yields in province *i* in year *t*,$\;m_{t}$ denotes the annual average monetary policy shock. $X_{it}$ deno*t*es province-specific information that affects the response of local government bonds to monetary policy shocks, which here refers to local government competition.

Following Miao et al. (2017) and Wang et al. (2023), local government competition indicator is constructed [40,41].Under the economic efficiency-oriented appraisal system, local governments tend to take neighboring regions and economically developed regions as crucial competitive controls, and actively attract liquid production factors by implementing a series of strategic competitive means, with a view to realizing rapid catch-up development of the economy, and then narrowing the development gap with these benchmark regions. The local government competition indicator not only takes into account the economic development of local governments in comparison with neighboring provinces, but also places it in the national context, so that it can comprehensively and objectively reflect the intensity of local government competition in each region. Its specific calculations are as follows:


$$\begin{array}{cc} & \text{Local government competition indicator }=\\ & \frac{\text{Highest GDP per capita in neighboring provinces other than the home province }}{\text{GDP per capita in the province }}\\ & \times \frac{\text{Highest GDP per capita in the country }}{\text{GDP per capita in the province}} \end{array}$$

When the coefficient of local government competition indicator is large, local economic development is relatively lagging behind, and competition is needed to narrow the gap with other regions; on the other hand, when the coefficient of local government competition is small, it means that the local economic development is better, and the gap with the reference region is smaller, and the pressure of competition is relatively small.

To account for the effects of provincial heterogeneity that does not vary over time, this paper incorporates provincial fixed effects into the regression model (2):


$$\begin{array}{c} \Delta y_{it}=\mu_{i}+\beta m_{t}+\gamma X_{it}+\delta (m_{t}\times X_{it})+\varepsilon_{it}\; \end{array}$$ (3)

As indicated by Table 4, the interaction term between monetary policy shocks and local governments is significantly negative both for yields to maturity and for credit spreads, suggesting that the more competitive local governments are, the less responsive local government bond yields and credit spreads are to monetary policy shocks. This implies that local financial frictions may be an important reason for the heterogeneity of local government responses. In Table 5, the interaction term after controlling for provincial fixed effects is also significantly negative, which further indicates that competition among local governments has a negative moderating effect.

**Table 4:** Interaction of local government competition.

	CB yields	CB credit spreads
MP shock	0.34 (0.21)	0.67 (0.42)
MP shock×LGC	−0.11 (0.02)	−0.21 (0.04)
N	450	450

Note: CB stands for chengtou bonds. MP shock stands for monetary policy shock. LGC stands for local government competition. Standard errors are reported in parentheses.

**Table 5:** Interaction of local government competition: Provincial Fixed-Effects Model.

	CB yields	CB credit spreads
MP shock	0.27 (0.15)	0.56 (0.27)
MP shock×LGC	−0.08 (0.01)	−0.14 (0.03)
FE	Yes	Yes
N	450	450

Note: CB stands for chengtou bonds. MP shock stands for monetary policy shock. LGC stands for local government competition. Standard errors are reported in parentheses. FE denotes the inclusion of fixed effects.

### Borrowing costs and local government behavior

To explore the impact of local government borrowing costs (yield to maturity) on local government behavior (fiscal policy), referring to Adelino et al. (2017) [42], we estimate the following regression equation:


$$\begin{array}{c} {log\;G}_{it}=\text{1}+\beta R_{it}+\Theta X_{it}+\varepsilon_{it} \end{array}$$ (4)

where $G_{it}$ indicates the investment expenditure of local governments or the scale of debt issuance, $R_{it}$ is the annual average of the yield to maturity of local government bonds, and here the annual summation of monetary policy shocks is used as its instrumental variable, $X_{it}$ includes the GDP of local governments, fiscal revenues, and yields to maturity of treasury bonds. To mitigate the problem of misleading regression resulting from common trends, we substitute the dependent variable in its level form with its growth rate, specifically ${log\;G}_{it}\;-\;{log\;G}_{it-\text{1}}$.

In order to better identify the impact of local government borrowing costs on government investment, this paper incorporates provincial fixed effects $\mu_{i}$ and time fixed effects $\lambda_{t}$ into regression model (4) to account for provincial characteristics that do not change over time and common temporal trends affecting all provinces (such as national economic cycles and policy environments):


$$\begin{array}{c} ogG_{it}=\mu_{i}+\lambda_{t}+\beta R_{it}+\theta X_{it}+\varepsilon_{it}\; \end{array}$$ (5)

Table 6 shows that local government bond yields have a significant impact on local government bond issuance and investment spending, with the impact on local government bond issuance being somewhat larger, more than twice the change in local government investment spending. A 1 percentage point decrease in the yield to maturity on local government bonds is associated with a 2.32 percentage point increase in local government investment spending and a 4.78 percentage point increase in the size of local government debt issuance, suggesting that local government fiscal policy (investment spending and the size of debt issuance) responds robustly to their cost of borrowing, which significantly influences the fiscal behavior of local governments. This further solidifies the logical chain of monetary policy influencing local government fiscal policy through borrowing costs, which is referred to in this paper as the local government debt channel of monetary policy. The regression results regarding growth rates in Table 6 also support this conclusion. This logical chain builds a solid empirical foundation for the model analysis below. Table 7 presents the regression results after incorporating provincial fixed effects and time fixed effects. The results indicate that local government bond yields have a significant impact on local government bond issuance and investment expenditure, thereby continuing to support the conclusion that local government bonds serve as a channel for monetary policy.

**Table 6:** Borrowing costs and local government behavior.

	LG investment	LGB issuance	LG investment growth	LGB issuance growth
LGB yields	−2.32 (0.34)	−4.78 (1.65)	−0.32 (0.03)	−0.89 (0.21)
F value	354.62	126.78	1109.23	807.42
N	450	450	420	420

Note: LGB stands for local government bonds, LG stands for local government. Standard errors are reported in parentheses. The F-values for all of the above models exceed the critical value of 19.38, indicating that the instrumental variables exhibit strong correlation and validity, and that the estimation results are reliable.

**Table 7:** Borrowing costs and local government behavior: Fixed-effects model.

	LG investment	LGB issuance
LGB yields	−2.03 (0.28)	−3.63 (0.92)
FE	Yes	Yes
F value	542.86	708.32
N	450	450

Note: LGB stands for local government bonds, LG stands for local government. Standard errors are reported in parentheses. FE indicates the inclusion of province-level fixed effects and time-period fixed effects. The F-values for all of the above models exceed the critical value of 19.38, indicating that the instrumental variables exhibit strong correlation and validity, and that the estimation results are reliable.

### Impulse response analysis of monetary policy shocks and local government behavior

Following Gertler and Karadi (2015) and Chen et al. (2023) [36,37], we use a Proxy SVAR model based on external instrumental variables to analyze the impact of monetary policy shocks on local government behavior in China. The data interval is from January 2009 to December 2023. We seasonally adjust the relevant data, which are obtained from the Wind database. We first consider the following lagged P-order VAR model:


$$\begin{array}{c} Y_{t}=a+\sum_{j=\text{1}}^{p}B_{j}Y_{t-j}+DX_{t}+A_{\text{0}}\varepsilon_{t} \end{array}$$ (6)

Where endogenous variables $Y_{t}$ include monetary policy instruments (R007), abbreviated as MP, local government fiscal policy variables (year-on-year growth rate of industrial value added, abbreviated as IP, year-on-year growth rate of commodity housing sales price, abbreviated as HP, and local government debt size, abbreviated as DB), CPI inflation, and credit spreads on chengtou bonds, abbreviated as CS. This paper draws on the methodology of Ramey (2011) and adopts the practice of substituting one variable at a time in the model to examine the substituted variable's impulse response to maintain sufficient degrees of freedom in the vector autoregressive model [43]. To control for external shocks, exogenous variables $X_{t}$ are added to the VAR, including the VIX index, the commodity price index, abbreviated as CP, and the one-year U.S. Treasury yield, abbreviated as US. High-frequency policy surprises are converted to monthly frequencies by summing the daily changes in the 7-day repo IRS.

The VAR residuals in reduced form $u_{t}$ is a linear combination of structural shocks $\varepsilon_{t}$, $u_{t}\;=\;A_{\text{0}}\varepsilon_{t}$, and hence the variance covariance matrix of the residuals in reduced form is$\;\Omega =A_{\text{0}}A_{\text{0}}$.’ Since we are only interested in the effects of monetary policy shocks, our goal is to identify the columns in that correspond to the contemporaneous effects of monetary policy shocks. We estimate the model on the sample space described above. The first stage regression is used to identify the contemporaneous impact of the policy shock. Once the contemporaneous impact is identified, the dynamic effects of the monetary policy shock are computed using the estimated VAR coefficients in reduced form.

It should be noted here that since China's monetary policy operates with a variety of policy instruments, this paper mainly uses the 7-day repo rate (R007) in the interbank market as the policy variable and the reserve ratio as an alternative variable for robust analysis. Since there is no monthly frequency indicator variable for local government fiscal policy, under the dual-wheel-driven development model of industrialization and urbanization, the data related to industrial value-added and house prices can reflect the behavioral logic of the local government.

**Fig 3:**
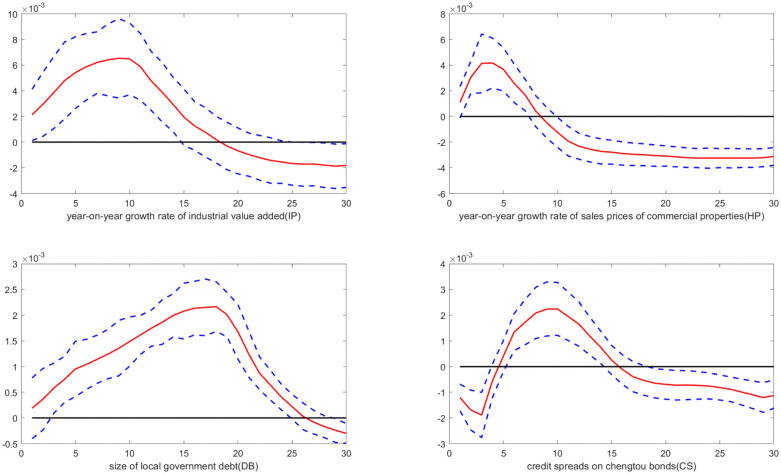
Impulse responses to monetary policy shocks. The blue dashed lines indicate a 68% confidence interval, and the red solid line indicates the impulse response of the variables of interest. We calculated the F-statistic for the first-stage regression using instrumental variables to be 98.34 (significantly greater than 10), indicating that there is no issue of weak instrumental variables, thereby validating the correlation of the instrumental variables.

Fig 3 shows that easing monetary policy shocks have a significant and persistent effect on local government’s behavior. Specifically, when the policy rate falls by 1 percentage point, it leads to a fall in local governments’ borrowing costs that lasts for a few months before rising a few months later. Simultaneously, the positive response of local governments’ debt size is larger and more persistent. In contrast, the rise in Year-on-year growth rate of industrial value added is more persistent and enters the negative space only after the 18th period. The persistence of the rise in the year-on-year growth rate of commercial property sales prices is relatively weak, lasting only seven periods. Overall, this VAR impulse response provides further empirical evidence for the local government debt channel of monetary policy and serves as a better guide for the model building and analysis that follows.

### Summary of the empirical analysis

The empirical section looks at how local government bonds are affected by monetary policy shocks. Local government bond rates to maturity typically respond by 0.24 percentage points to a monetary policy shock of 1 percentage point; this dampened reaction is less than that of the underlying corporate bonds. According to provincial estimates, this limited response exhibits cross-space heterogeneity. This diverse response may be directly linked to local government competition, according to additional analysis, indicating that local financial frictions play a significant role in shaping local government responses. The empirical analysis of borrowing costs and local government behavior provides a further realistic basis for the local government debt channel of monetary policy. The Proxy SVAR model's impulse response analysis demonstrates how monetary policy shocks can impact local fiscal policy by influencing the cost of borrowing for local governments. China has a local government debt channel for monetary policy, according to both regression and VAR research.

## Theoretical models

Based on the empirical evidence presented in the previous section, this section constructs a two-region open economy model that incorporates the local government bond channel of monetary policy, i.e., the response of local government borrowing costs to monetary shocks, the strength of which affects the transmission effect of monetary policy shocks to the real economy. We distinguish between local and central government fiscal policy and additionally include the central government that receives tax revenues through tax sharing to finance transfers to local governments. The central bank chooses the risk-free rate. Each region includes the productive sector, households, banks, and local government. The productive sector produces heterogeneous intermediate goods and packages the intermediate goods into final goods for consumption by households and the central government in the two regions, and for investment by the producers of investment goods, while the local government uses only locally produced goods for consumption and investment. Households in each region consume, save, and provide labor. Financial institutions in each region purchase corporate and local government bonds in both regions and take deposits from households. Local governments are financed by corporate tax revenues, bond issues, and central government transfers, which are used for local government consumption and investment.

This model can quantify the effects of local financial frictions on the response of local government finance to macroeconomic shocks, which in this case manifest as a dampened monetary policy response as previously described. The excess spread between the risk-free rate and local government borrowing costs depends on the risk-free rate, the deviation of debt from steady state, and the parameters of the relationship between monetary policy shocks and the interest rate actually paid by local governments (the coefficients in the previous regression). It also captures potential sources of heterogeneity in the transmission of monetary policy in China.

### Household

Representative households maximize expected intertemporal utility:


$$\begin{array}{c} E_{\text{0}}\left\{\sum_{t=\text{0}}^{\infty }[\frac{{\left(c_{t}-\psi c_{t-\text{1}}\right)}^{\text{1}-\sigma_{c}}}{\text{1}-\sigma_{c}}-\chi \frac{l_{t}^{\text{1}+\sigma_{l}}}{\text{1}+\sigma_{l}}]\right\} \end{array}$$ (7)

where $c_{t}$i s composite consumption, $l_{t}$ is the number of hours worked. Household has consumption habits, and his real budget constraint is:


$$\begin{array}{c} {b_{t}^{i}+d}_{t}+c_{t}=\frac{R_{t-\text{1}}^{d}d_{t-\text{1}}}{\pi_{t}}+w_{t}l_{t}+\Pi_{t}^{f}+div_{t}-x+\frac{R_{t-\text{1}}^{d}b_{t-\text{1}}^{i}}{\pi_{t}} \end{array}$$ (8)

where $w_{t}\;$ represents real wages. Households make one-year savings deposits with financial intermediaries. $d_{t}$ denotes the amount of the household's savings deposit, $R_{t-\text{1}}^{d}$ denotes the total nominal interest rate on deposits between period t-1 and period t, which has been set in period t-1. $b_{t}^{i}$ is a nominal single-tranche bond traded between two districts at the deposit rate common to all districts, which is the same as the rate set by the central bank. In addition, households receive profits $\Pi_{t}^{f}$ from firm ownership and dividends $div_{t}$ from financial intermediaries. Households make fixed transfers to the new financial intermediary during each period, which we represent by *x*. We define $\Lambda_{t,t+\text{1}}=U_{c,t+\text{1}}/U_{c,t}$.

The composite consumption $c_{t}$ aggregates the consumption sub-baskets for Regions 1 and 2, $c_{\text{1},t}$ and $c_{\text{2},t}$, which are summed using the Armington form:


$$\begin{array}{c} c_{t}={\left[\alpha_{\text{1}}^{\frac{\text{1}}{\phi }}{\left(c_{\text{1},t}\right)}^{\frac{\phi -\text{1}}{\phi }}+{\left(\text{1}-\alpha_{\text{1}}\right)}^{\frac{\text{1}}{\phi }}{\left(c_{\text{2},t}\right)}^{\frac{\phi -\text{1}}{\phi }}\right]}^{\frac{\phi }{\phi -\text{1}}} \end{array}$$ (9)

where the parameter ϕ represents the elasticity of substitution between regional goods and $\alpha_{\text{1}}$ is the relative preference of households for region 1 goods. Let $\rho_{\text{1},t}\equiv P_{\text{1},t}/P_{t}$ and $\rho_{\text{2},t}\equiv P_{\text{2},t}/P_{t}$ denote the real prices of region 1 and region 2 commodities measured in region 1 consumption units, respectively. The aggregate price index is


$$\begin{array}{c} P_{t}={\left[\alpha_{\text{1}}{\left(P_{\text{1},t}\right)}^{\text{1}-\phi }+(\text{1}-\alpha_{\text{1}}){\left(P_{\text{2},t}\right)}^{\text{1}-\phi }\right]}^{\text{1}/(\text{1}-\phi )} \end{array}$$ (10)

The sub-baskets $c_{\text{1},t}$ aggregates the differentiated consumption varieties $c_{\text{1},t}(j)$ in region 1 and $c_{\text{2},t}$ aggregates the differentiated consumption varieties $c_{\text{2},t}(j)$ in region 2, given by the following equation


$$\begin{array}{c} c_{\text{1},t}={\left[\int_{\text{0}}^{\text{1}}c_{\text{1},t}(j{)}^{(\theta -\text{1})/\theta }dj\right]}^{\theta /(\theta -\text{1})} \end{array}$$ (11)

$$\begin{array}{c} c_{\text{2},t}={\left[\int_{\text{0}}^{\text{1}}c_{\text{2},t}(j{)}^{(\theta -\text{1})/\theta }dj\right]}^{\theta /(\theta -\text{1})} \end{array}$$ (12)

where θ is the elasticity of substitution between goods within regions.

### Production

Following Sims and Wu (2021) and Gertler and Karadi (2011) [44,45], this model contains three different types of firms. The representative wholesale firm issues bonds to finance capital purchases and uses its own capital and labor to make goods. While representative investment goods generating enterprises create new investment goods utilizing final products, retail firms repackage wholesale commodities for resale and are subject to price stickiness.

Investment Goods Firms. Competitive investment goods producing firms use final goods to produce new investment goods $I_{t}^{w}$ and sell them to wholesale firms at price $p_{t}^{k}$. The investment goods producing firms use commodities from Regions 1 and 2 (in the same Armington form as the consumer goods aggregates) to obtain a composite investment good $I_{t}$, which in turn is used to produce new capital $I_{t}^{w}$.

The new investment goods production function is


$$\begin{array}{c} I_{t}^{w}=(\text{1}-S(\frac{I_{t}}{I_{t-\text{1}}}))I_{t} \end{array}$$ (13)

where S(·) is the investment adjustment cost function. Therefore, the firm's optimization problem is given by the following equation


$$\begin{array}{c} max\sum_{t=\text{0}}^{\infty }E_{\text{0}}\left[\Lambda_{\text{0},t+\text{1}}\left(p_{t}^{k}(\text{1}-S(\frac{I_{t}}{I_{t-\text{1}}}))I_{t}-I_{t}\right)\right] \end{array}$$ (14)

Wholesale Firms. A representative wholesale company produces output according to the following formula


$$\begin{array}{c} y_{t}^{w}=A_{t}l_{t}^{\text{1}-\alpha }K_{t-\text{1}}^{\alpha }{\left(K_{t-\text{1}}^{g}\right)}^{\alpha_{g}} \end{array}$$ (15)

where $A_{t}$ is exogenous productivity. $K_{t-\text{1}}^{g}$ denotes the public capital provided by the local government. $\alpha_{g}$ is the output elasticity with respect to public capital, which determines the productivity of public capital. $K_{t}\;$ is private capital owned by wholesale firms and evolves according to the standard law of motion:


$$\begin{array}{c} K_{t}=I_{t}^{w}+(\text{1}-\delta )K_{t-\text{1}} \end{array}$$ (16)

Following Sims and Wu (2021) [44], we assume that the wholesale firm faces an advance loan constraint and must issue perpetual bonds to finance a small portion of the new physical capital. The advance loan constraint is expressed as follows:


$$\begin{array}{c} \eta^{I}p_{t}^{k}I_{t}^{w}\leq Q_{t}^{f}\left(f_{t}-\kappa^{f}\frac{f_{t-\text{1}}}{\pi_{t}}\right) \end{array}$$ (17)

which $f_{t}$ represents the number of corporate bonds. Wholesale firms make decisions about labor, investment, and bond issuance to maximize the present value of their profits,


$$\begin{array}{c} max\sum_{t=\text{0}}^{\infty }E_{\text{0}}[\Lambda_{\text{0},t+\text{1}}\left(p_{t}^{w}y_{t}^{w}\left(\text{1}-\tau^{i}\right)-w_{t}l_{t}-p_{t}^{k}I_{t}^{w}-\frac{f_{t-\text{1}}}{\pi_{t}}+Q_{t}^{f}\left(f_{t}-\kappa^{f}\frac{f_{t-\text{1}}}{\pi_{t}}\right))\right] \end{array}$$ (18)

subject to the production function (15), the capital accumulation equation (16), and the advance loan constraint (17).

Retail firms. The retail firm *h* repackages the wholesale product, $y_{t}(h)=y_{t}^{w}(h)$, and sells it at a price $P_{\text{1},t}(h)$. The firm faces a Rotemberg price adjustment cost, and the cost function can be expressed by $\frac{\omega^{p}}{\text{2}}{\left(\frac{P_{\text{1},t}(h)}{P_{\text{1},t-\text{1}}(h)}\frac{\text{1}}{\pi^{\text{1}}}-\text{1}\right)}^{\text{2}}P_{\text{1},t}y_{t}$. The retail firm *h* chooses its price to optimize real profit, given by the following equation:


$$\begin{array}{c} \sum_{k=\text{0}}^{\infty }E_{t}\left[\Lambda_{t,t+k}\left(\frac{P_{\text{1},t+k}(h)}{P_{t+k}}y_{t+k}(h)-p_{t+k}^{w}y_{t+k}(h)-\frac{\psi }{\text{2}}{\left(\frac{P_{\text{1},t+k}(h)}{P_{\text{1},t+k-\text{1}}(h)}\frac{\text{1}}{\pi^{\text{1}}}-\text{1}\right)}^{\text{2}}{\frac{P_{\text{1},t+k}(h)}{P_{t+k}}y}_{t+k}\right)\right] \end{array}$$ (19)

After realizing equilibrium, optimal price setting implies


$$\begin{array}{c} \frac{p_{t}^{w}}{\rho_{\text{1},t}}=\frac{\theta -\text{1}}{\theta }+\frac{\omega^{\text{p}}}{\theta }(\frac{\pi_{t}^{\text{1}}}{\pi^{\text{1}}}-\text{1})\frac{\pi_{t}^{\text{1}}}{\pi^{\text{1}}}-\frac{\omega^{\text{p}}}{\theta }E_{t}\Lambda_{t,t+\text{1}}(\frac{\pi_{t+\text{1}}^{\text{1}}}{\pi^{\text{1}}}-\text{1})\frac{\pi_{t+\text{1}}^{\text{1}}}{\pi^{\text{1}}}\frac{y_{t+\text{1}}}{y_{t}} \end{array}$$ (20)

### Financial intermediation

There is a continuum of financial intermediaries on the interval, much like Sims and Wu (2021) and Bi and Traum (2023) [44,46]. The equal number of new intermediaries that get start-up funding from households replace the small percentage of intermediaries that randomly leave throughout each period. Net worth is returned to households by financial intermediaries, who build up net worth until they leave.

Financial intermediary *j* can purchase local government bonds $b_{\text{1},t}^{j}\;$ and corporate bonds $f_{\text{1},t}^{j}$ in its region or from region 2,$\;b_{\text{2},t}^{j}$ and $f_{\text{2},t}^{j}$, and its purchases are financed by household deposits $d_{t}^{j}$ and net worth $n_{t}^{j}$ in its region, with the real balance sheet given by the following equation


$$\begin{array}{c} Q_{t}f_{\text{1},t}^{j}+b_{\text{1},t}^{j}+Q_{t}^{*}f_{\text{2},t}^{j}+b_{\text{2},t}^{j}=d_{t}^{j}+n_{t}^{j} \end{array}$$ (21)

The equation for the evolution of the net worth of financial intermediary *j* is


$$\begin{array}{c} n_{t}^{j}=(R_{t}^{b}-R_{t-\text{1}}^{d})\frac{b_{\text{1},t-\text{1}}^{j}}{\pi_{t}}+(R_{t}^{f}-R_{t-\text{1}}^{d})\frac{Q_{t}f_{\text{1},t-\text{1}}^{j}}{\pi_{t}}+(R_{t}^{b*}-R_{t-\text{1}}^{d})\frac{b_{\text{2},t-\text{1}}^{j}}{\pi_{t}} \end{array}$$

$$\begin{array}{c} \;+(R_{t}^{f*}-R_{t-\text{1}}^{d})\frac{Q_{t}^{*}f_{\text{2},t-\text{1}}^{j}}{\pi_{t}}-R_{t-\text{1}}^{d}\frac{n_{t-\text{1}}^{j}}{\pi_{t}} \end{array}$$ (22)

where $R_{t}^{b}-R_{t-\text{1}}^{d}$, $R_{t}^{f}-R_{t-\text{1}}^{d}$, $R_{t}^{b*}-R_{t-\text{1}}^{d}$, $R_{t}^{f*}-R_{t-\text{1}}^{d}$, respectively, are the excess returns of financial intermediaries in Region 1 holding local government bonds as well as corporate bonds compared to deposit rates. The return on holding corporate bonds is


$$\begin{array}{c} {\;{\;R}_{t}^{f}=\frac{\text{1}+\kappa^{f}Q_{t}}{Q_{t-\text{1}}},R}_{t}^{f*}=\frac{\text{1}+\kappa^{f*}Q_{t}^{*}}{Q_{t-\text{1}}^{*}} \end{array}$$ (23)

Each period a fraction 1−σ of financial intermediary randomly exits and returns its net worth to the household owner, and the financial intermediary *j* maximizes its lifetime discounted net worth


$$\begin{array}{c} maxV_{t}^{j}=(\text{1}-\sigma )E_{t}\Lambda_{t,t+\text{1}}n_{t+\text{1}}^{j}+\sigma \beta E_{t}\Lambda_{t,t+\text{1}}V_{t+\text{1}}^{j} \end{array}$$ (24)

An intermediary will desire to increase its assets indefinitely by accepting household deposits if investing in municipal bonds may yield positive excess returns.In order to restrict their capacity to do so, we presume that financial intermediaries encounter a CSV issue, as shown in Gertler and Karadi (2011): at the end of a period, the intermediary can transfer a portion of its assets and pass them on to the household owner, in which case the depositor can reclaim the remaining assets and force the intermediary to go bankrupt [45]. Following Krenz (2022) [47], the incentive constraint is a CES combination of region 1 and region 2 assets:


$$\begin{array}{c} V_{t}^{j}\geq \eta^{\nu }(m_{t}^{f,j}+\theta^{b}m_{t}^{b,j}) \end{array}$$ (25)

where


$$\begin{array}{c} \;{\;m}_{t}^{b,j}={\left[\gamma_{b}^{\frac{\text{1}}{\sigma_{b}}}{\left(b_{\text{1},t}^{j}\right)}^{\frac{\sigma_{b}-\text{1}}{\sigma_{b}}}+{\left(\text{1}-\gamma_{b}\right)}^{\frac{\text{1}}{\sigma_{b}}}{\left(b_{\text{2},t}^{j}\right)}^{\frac{\sigma_{b}-\text{1}}{\sigma_{b}}}\right]}^{\frac{\sigma_{b}}{\sigma_{b}-\text{1}}} \end{array}$$ (26)

$$\begin{array}{c} \;m_{t}^{f,j}={\left[\gamma_{f}^{\frac{\text{1}}{\sigma_{f}}}{\left(Q_{t}f_{\text{1},t}^{j}\right)}^{\frac{\sigma_{f}-\text{1}}{\sigma_{f}}}+{\left(\text{1}-\gamma_{f}\right)}^{\frac{\text{1}}{\sigma_{f}}}{\left(Q_{t}^{*}f_{\text{2},t}^{j}\right)}^{\frac{\sigma_{f}-\text{1}}{\sigma_{f}}}\right]}^{\frac{\sigma_{f}}{\sigma_{f}-\text{1}}}\; \end{array}$$ (27)

The parameters $\sigma_{b}$ and $\sigma_{f}$ denote the interest rate elasticity of demand for assets, $\gamma_{b}$ and $\gamma_{f}$ denote the local bias of asset holdings. The incentive constraint's assumption of imperfect substitution between Region 1 and Region 2 assets can represent the varying asset-type preferences of financial intermediaries, their different views on cross-region asset risk, and the convenience gains brought about by inter-region regime changes [47,48]. The incentive constraint implies that if a financial intermediary chooses to go bankrupt, it can retain a portion of its corporate bond portfolio and also a portion of its local government bond portfolio. Assumption $\text{0}\leq \theta^{b}\leq$1, this suggests that corporate bonds can be transferred more easily via financial intermediaries than bonds issued by local governments. The above condition implies that the value of continuing to act as an intermediary should be greater than or equal to the amount of money that the financial intermediary $V_{t}^{j}$ can transfer. The parameter $\eta_{t}^{\nu }$ reflects the tightness of the credit market: higher the parameter $\eta_{t}^{\nu }$ is, the more money the financial intermediary can transfers, and the less willing savers are to lend money.

Finally, we assume that new entrant intermediaries receive start-up capital from households, which is denoted by 𝑥. The equation for the change in total net worth of the financial institutions sector is:


$$\begin{array}{c} \begin{array}{c} n_{t}=\sigma [(R_{t}^{b}-R_{t-\text{1}}^{d})\frac{b_{\text{1},t-\text{1}}}{\pi_{t}}+(R_{t}^{f}-R_{t-\text{1}}^{d})\frac{Q_{t-\text{1}}f_{\text{1},t-\text{1}}}{\pi_{t}}]\\ +\sigma [(R_{t}^{b*}-R_{t-\text{1}}^{d})\frac{b_{\text{2},t-\text{1}}}{\pi_{t}}+(R_{t}^{f*}-R_{t-\text{1}}^{d})\frac{Q_{t-\text{1}}^{*}f_{\text{2},t-\text{1}}}{\pi_{t}}]\\ +\sigma R_{t-\text{1}}^{d}\frac{n_{t-\text{1}}}{\pi_{t}}+(\text{1}-\sigma )x \end{array} \end{array}$$ (28)

Given that all financial intermediary makes the same best choices, the financial intermediary sector's balance sheet is


$$\begin{array}{c} b_{\text{1},t}+Q_{t}f_{\text{1},t}+b_{\text{2},t}+Q_{t}^{*}f_{\text{2},t}=d_{t}+n_{t} \end{array}$$ (29)

### Local government

We assume that government consumption and investment are fully biased in favor of intra-regional goods, so that they now buy local goods at the prices $\rho_{\text{1},t}$. In order to pay for its public spending, the local government issues bonds, receives transfers from the federal government, and collects business tax. The following formula provides its actual financial constraint:


$$\begin{array}{c} \rho_{\text{1},t}g_{t}^{i}+{\rho_{\text{1},t}g}_{t}^{c}=\mu \tau_{t}+b_{t}^{G}-(\text{1}+r_{t}^{G})b_{t}^{G}+{tr}_{t} \end{array}$$ (30)

where $g_{t}^{i}$ denotes local government public investment and $g_{t}^{c}$ denotes local government public consumption. The public consumption expenditure of the local government is exogenously given, $g_{t}^{c}=\alpha_{gc}g$, where g denotes the total steady state expenditure of the local government, $\alpha_{gc}\;$ is the ratio of public consumption expenditure of the local government to the total expenditure. μ is represents the local government tax revenue share ratio, $\tau_{t}$ is the tax revenue,${\;\tau_{t}\;=p}_{t}^{w}y_{t}^{w}\tau^{i}$, and ${tr}_{t}$ is the transfer payment from the central government to the local government. $b_{t}^{G}$ denotes the bonds issued by the local government, $r_{t}^{G}$ is the local government bond interest rate, note that the local government bond interest rate is assumed to be independent of the household savings interest rate here, and following Schmitt-Grohe and Uribe (2003) [49], the local government bond interest rate is determined by the following equation


$$\begin{array}{c} r_{t}^{G}=r^{*}+\theta^{G}(r_{t}^{d}-r^{*})+\phi^{G}(e^{b_{t}^{G}-b^{G}}-\text{1}) \end{array}$$ (31)

where the imperfect response of the government bond rate to the risk-free rate is captured by the parameter $\theta^{G}$, which is the response parameter of local government bond yields to monetary policy shocks, a formulation that has the advantage of allowing for the setting of an arbitrary steady-state level of debt and capturing local financial frictions more easily.

When local governments issue bonds, they must consider their impact on current and future borrowing costs, which the above setting takes into account. The impact of local government bond issuance on borrowing rates should not be disregarded, and it influences both the model's steady state and how debt responds to short-term shocks. If the level of local government bonds did not affect their borrowing costs, then at normal interest rate levels the government would accumulate an infinitely large level of debt. The market's reaction to excessive local government debt would be unreasonably understated if the impact of debt on borrowing costs were not taken into account.

Following Ai and Wang (2021) [50], local governments are assumed to choose their own public investment expenditures to maximize their own utility under the constraint of the following objective function, which is given by the following equation


$$\begin{array}{c} E_{t}\left\{\sum_{s=t}^{\infty }\beta_{g}^{s-t}(\varepsilon_{g,t}ln(y_{t}-y_{t-\text{1}}-\varphi_{y}^{*}(y_{t}^{*}-y_{t-\text{1}}^{*})+\text{1})+\phi_{b,t})\right\} \end{array}$$ (32)

where $\varepsilon_{g,t}\;$ denotes a positive intertemporal preference shock for local governments, $\varphi_{y}^{*}$ denotes that local governments face additional smoothing incentives, and $\varphi_{y}^{*}$ is taken to be 0 or 1, which indicates whether the local government's objectives include comparisons with other regions (i.e., whether or not there is competition for local governments). The current “dual incentive” hypothesis regarding local government behavior—growth-oriented incentives (“competing for growth”) [2] and stability- and sustainability-oriented incentives (“aversion to volatility”) [31]—is taken into consideration in the specification of the local government objective function in this paper. Additionally, this framework provides the microfoundation for the “local government bond channel” to produce distinct macroeconomic effects by enabling local governments to react to macroeconomic shocks (like monetary policy shocks) differently than central-level government. Local governments will also be concerned about fluctuations in the economy, i.e., the


$$\begin{array}{c} \phi_{b,t}=-\varphi_{b}(\frac{b_{t}^{G}-b^{G}}{b^{G}})-\varphi_{y}(\frac{y_{t}-y_{t-\text{1}}}{y}) \end{array}$$ (33)

where $\varphi_{b}$ and $\varphi_{y}$ represent the coefficients of government utility in response to bond and output fluctuations, the above equation shows that local governments generate additional negative utility due to rising local government debt levels and economic fluctuations, i.e., the growth motive and the stability motive in local government utility.

Local government’s public investment $g_{t}^{i}$ becomes productive public capital after a period of time and affects the productivity of firms, the equation of motion of local government public capital is $K_{t}^{G}=(\text{1}-\delta_{G})K_{t-\text{1}}^{G}+g_{t}^{i}$.

In the local government optimization process, all equations (including the relevant equations of households, vendors, financial intermediaries and the central government) become the constraint equations for local government decision-making, and the local governments of the two regions play the game and generate the Nash equilibrium based on the observation of the whole economic operation. For the solution method, see Bodenstein et.al (2019) [51].

### Central government and monetary policy

A monetary union establishes a common monetary policy for both regions by having the central bank set nominal interest rates across the economy using the Taylor rule:


$$\begin{array}{c} \text{ln}\frac{R_{t}^{d}}{R^{d}}=\rho_{R}\text{ln}\frac{R_{t-\text{1}}^{d}}{R^{d}}+(\text{1}-\rho_{R})(\phi_{\pi }\text{ln}\frac{\pi_{t}^{ag}}{\pi^{ag}}+\phi_{y}\text{ln}\frac{y_{t}^{ag}}{y^{ag}})+\varepsilon_{t}^{R} \end{array}$$ (34)

The monetary authority responds to changes in the average consumer price inflation, $ln\frac{\pi_{t}^{ag}}{\pi^{ag}}=\text{0}.\text{5}ln\frac{\pi_{t}}{\pi }+\text{0}.\text{5}ln\frac{\pi_{t}^{*}}{\pi^{*}}$, and the output-weighted average, $ln\frac{y_{t}^{ag}}{y^{ag}}=\text{0}.\text{5}ln\frac{y_{t}}{y}+\text{0}.\text{5}ln\frac{y_{t}^{*}}{y^{*}}\;$.

The central government carries out government expenditures and transfers, and its revenues are derived from the share of tax revenues:


$$\begin{array}{c} g_{t}^{cen}+{tr}_{t}+tr_{t}^{*}=(\text{1}-\mu )(\tau_{t}+\tau_{t}^{*}) \end{array}$$ (35)

where $g_{t}^{cen}$ denotes central government expenditure. To simplify the analysis, it is assumed that the level of central government expenditure is equally distributed between the two regions.

### Market clearing

The clearing of the corporate bond market means that


$$\begin{array}{c} f_{t}=f_{\text{1},t}+rer_{t}f_{\text{1},t}^{*} \end{array}$$ (36)

$$\begin{array}{c} f_{t}^{*}=\frac{f_{\text{2},t}}{rer_{t}}+f_{\text{2},t}^{*} \end{array}$$ (37)

The clearing of the local government bond market means that


$$\begin{array}{c} b_{t}^{G}=b_{\text{1},t}+rer_{t}b_{\text{1},t}^{*} \end{array}$$ (38)

$$\begin{array}{c} b_{t}^{G*}=\frac{b_{\text{2},t}}{rer_{t}}+b_{\text{2},t}^{*} \end{array}$$ (39)

Cross-regionally traded bond market clearing:


$$\begin{array}{c} b_{t}^{i}+{rer}_{t}b_{t}^{i*}=\text{0} \end{array}$$ (40)

Product market clearing:


$$\begin{array}{c} y_{t}=\alpha_{\text{1}}\rho_{\text{1}t}^{-\phi }(c_{t}+\;I_{t})+(\text{1}-\alpha_{\text{1}}^{*}){\left(\rho_{\text{1},t}/{rer}_{t}\right)}^{-\phi^{*}}{(c}_{t}^{*}+I_{t}^{*})+g_{t}^{i}+g_{t}^{c} \end{array}$$ (41)

$$\begin{array}{c} y_{t}^{*}=\alpha_{\text{1}}^{*}{\left(\rho_{\text{1},t}^{*}\right)}^{-\phi^{*}}(c_{t}^{*}+\;I_{t}^{*})+(\text{1}-\alpha_{\text{1}}){\left(\rho_{\text{1},t}^{*}{\;rer}_{t}\right)}^{-\phi }(c_{t}+\;I_{t})+g_{t}^{i*}+g_{t}^{c*} \end{array}$$ (42)

Given that the nominal exchange rate remains unchanged, the condition that the real exchange rate is correlated with relative inflation can be used to characterize the adjustment of regional relative prices:


$$\begin{array}{c} er_{t}/rer_{t-\text{1}}=\pi_{t}^{*}/\pi_{t} \end{array}$$ (43)

The net foreign asset equation is expressed as follows:


$$\begin{array}{c} \begin{array}{c} b_{t}^{i}+b_{\text{2},t}+Q_{t}^{*}f_{\text{2},t}-b_{\text{1},t}^{*}{rer}_{t}-Q_{t}f_{\text{1},t}^{*}{rer}_{t}\\ \;=\frac{R_{t-\text{1}}^{d}b_{t-\text{1}}^{i}}{\pi_{t}}+\frac{R_{t}^{b*}b_{\text{2},t-\text{1}}}{\pi_{t}}+\frac{R_{t}^{f*}Q_{t-\text{1}}^{*}f_{\text{2},t-\text{1}}}{\pi_{t}}-\frac{R_{t}^{f}Q_{t-\text{1}}f_{\text{1},t-\text{1}}^{*}{rer}_{t-\text{1}}}{\pi_{t}}\\ \;-\frac{R_{t}^{b}b_{\text{1},t-\text{1}}^{*}{rer}_{t-\text{1}}}{\pi_{t}}+\rho_{\text{1},t}(c_{\text{1},t}^{*}+i_{\text{1},t}^{*})-\rho_{\text{2},t}^{*}{rer}_{t}(c_{\text{2},t}+i_{\text{2},t}). \end{array} \end{array}$$ (44)

## Quantitative analysis

In this section, the significance of the local government debt channel of monetary policy is examined using numerical simulations of the study once the parameters in the theoretical model have been assigned values. The variation in ow local government bond rates react to monetary policy shocks suggests that monetary policy has a varied effect on local government fiscal policy, which depends on regional financial frictions that considerably reduce the impact of monetary policy.

### Parameter calibration

In this paper, the parameters in the model are calibrated with reference to existing literature or data. The parameters to be calibrated mainly include various structural parameters and steady-state parameters. The structural parameters mainly refer to the existing literature, while the steady-state parameters are mainly calibrated based on historical data during the sample period or based on the steady-state relational equation. Following Zhu and Xu (2018) and Mei et al. (2018) [31,52], the intertemporal elasticity of labor $\sigma_{l}$ is set to 1, and the intertemporal elasticity of substitution of consumption $\sigma_{c}$ is set to 1, and the household utility discounting parameter β is set to 0.99. Following Ai and Wang (2021) [50], the government utility discounting parameter $\beta_{g}$ is set to 0.99, same as that for households. Following Lu and Huang (2011) [53], the consumption habit parameter ψ is set to 0.8. In order to achieve a steady-state price markup of 10%, the intra-area elasticity of substitution of goods θ is set to 11. Following Bi and Traum (2023) [46], the price adjustment cost parameter $\omega^{p}\;$is calibrated to a value that corresponds to the probability that firms cannot adjust their prices in the Calvo model of 0.75. In line with the vast majority of china’s economy’s literature, both the depreciation rate of firm capital δ and the depreciation rate of public capital $\delta_{g}$ are set to 0.25, i.e., capital is assumed to have a useful life of 10 years. According to Liu (2018) [54], the output elasticity of public capital $\alpha_{g}$ is set to 0.2. The degree of preference for foreign goods in the region $\alpha_{\text{1}}$ is set to 0.3, i.e., the regional preference is 0.7. Following Ai and Wang (2021) [50], the output elasticity of private capital α is set to 0.45. According to Nakamura and Steinsson (2014) [13], the elasticity of substitution between goods is is set to 2. Referring to Hao et al. (2020) [55], the investment adjustment cost parameter ϕ is set to 5. The steady state value of labor supply time is set to 1, and the value of parameter χ can be obtained by model calculation.

The relevant local government parameters are calibrated based on local government revenue and expenditure data for the period from 2004 to 2019. The regional competition parameter $\varphi_{y}^{*}$ is either 1 or 0. A value of 1 for $\varphi_{y}^{*}$ indicates that there is regional scale competition among local governments, and a value of 0 for $\varphi_{y}^{*}$ indicates that there is no regional scale competition among local governments. The coefficient of government bond interest rate response to monetary policy $\theta^{G}$ is obtained from the above empirical evidence and is set to 0.24. Following Schmitt and Uribe (2003) [49], the interest rate elasticity of government debt $\phi^{G}$ is set to 0.0000335. Based on the data for the sample period, the mean value of the share of China's local government expenditures in GDP is 0.20. The quarterly spread on local government debt yields is set at 0.6%, based on the average spread on municipal bonds during the sample period. Based on the realistic average tax rate calculated by Shi et al. (2019) [56], the revenue tax rate $\tau^{i}$ is set to 0.12. According to the measurements of Lv et al. (2021) [57], the tax share ratio μ is set to 0.5. Following Ai and Wang (2021) [50], the ratio of the steady-state value of the stock of local government bonds to local output is set to 0.6. Based on historical data, the local government expenditure as a proportion of local output is set to the mean value of the ratio of local government expenditure to local output is 17%, and the ratio of government consumption expenditure to government expenditure $\alpha_{gc}$ is set to 1/3, i.e., the ratio of investment expenditure to local output is 2/3, and the mean value of central government expenditure to GDP is 4.1%.

Parameters related to the corporate bond market and financial intermediaries. Following Poutineau and Vernandel (2015) [58], the interest rate elasticity of demand for assets $\sigma_{b}$ and $\sigma_{f}$ is set to −2, and the local preference of asset holdings ${\;\gamma }_{b}$ and $\gamma_{f}$ is set to 0.7, reflecting the high degree of integration between China’s regions. Following Sims and Wu (2021) [44], the ratio of corporate debt to annualized GDP is set to 1.5 based on data for the sample period. The average maturity of corporate bonds is set to 5 years based on the most common five-year maturity in the corporate bond market, which translates into an attenuation coefficient $\kappa^{f}$ of 0.95 for corporate bonds. The proportion of wholesale firms that must invest through debt financing $\eta^{I}$ is set to 0.86. Based on the average spread on domestic five-year corporate bonds over the sample period, the yield spread on corporate assets owned by the banking sector is set at 0.8%. The steady state value of the yield spread for the financial sector determines the value of the financial friction parameter $\eta^{\nu }$ and $\theta^{b}$. According to Gertler and Karadi (2011) [45], the probability of survival of financial intermediaries σ is set to 0.95. Following He and Krishnamurthy (2019) [59], the steady state value of leverage of the financial sector is set to 3. The parameter value of start-up capital for new financial intermediaries *x* is chosen to satisfy the leverage of financial intermediaries.

The smoothing parameter for monetary policy is set to 0.8, the coefficient of response to inflation is set to 1.5, and the coefficient of response to output is set to 0.15. The parameters of the productivity shock process are directly referenced to the estimates of Zhu et al. (2018) [31], with the smoothing parameter set to 0.74 and the standard deviation of the shocks to 0.0054. The smoothing parameter for the other shocks is set to 0.8, with the standard deviation set to 0.01 (Table 8).

**Table 8:** Parameter values.

Parameters	Description	Value
β	Household utility discounting parameter	0.99
$\sigma_{l}$	Inter-temporal elasticity of labor	1
$\sigma_{c}$	Inter-temporal elasticity of substitution of consumption	1
$\beta_{g}$	Government utility discounting parameter	0.99
ψ	Consumption habit parameter	0.8
θ	Infra-regional elasticity of substitution of goods	11
$\omega^{p}$	Price adjustment cost parameter	128.16
δ	depreciation rate of firm capital	0.25
$\delta_{g}$	depreciation rate of public capital	0.25
$\alpha_{g}$	output elasticity of public capital	0.2
$\alpha_{\text{1}}$	degree of preference for foreign goods in the region 1	0.3
α	output elasticity of private capital	0.45
ϕ	the elasticity of substitution between goods	2
$\omega^{I}$	the investment adjustment cost parameter	5
χ	Relative utility weight of labor	3.83
$\varphi_{y}^{*}$	regional competition parameter	1
$\theta^{G}$	coefficient of government bond interest rate response to monetary policy	0.24
$\phi^{G}$	interest rate elasticity of government debt	0.00003
$\tau^{i}$	revenue tax rate	0.12
*u*	tax share ratio	0.5
$\alpha_{gc}$	ratio of consumption expenditure to total government expenditure	0.33
$\sigma_{b}$	Interest rate elasticity of demand for local government bonds	−2
$\sigma_{f}$	Interest rate elasticity of demand for firm bonds	−2
$\gamma_{b}$	Local preference of local government bond holdings	0.7
$\gamma_{f}$	Local preference of firm bond holdings	0.7
$\kappa^{f}$	attenuation coefficient for corporate bonds	0.95
$\eta^{I}$	proportion of wholesale firms that must invest through debt financing	0.86
$\eta^{\nu }$	Recoverability parameter	0.47
$\theta^{b}$	Government bond recoverability	0.88
σ	probability of survival of financial intermediaries	0.95
*x*	start-up capital for new financial intermediaries	11.55
$\rho_{R}$	smoothing parameter for monetary policy	0.8
$\phi_{\pi }$	coefficient of response to inflation	1.5
$\phi_{y}$	coefficient of response to output	0.15

### Dynamic effects of expansionary monetary policy shocks

**Fig 4:**
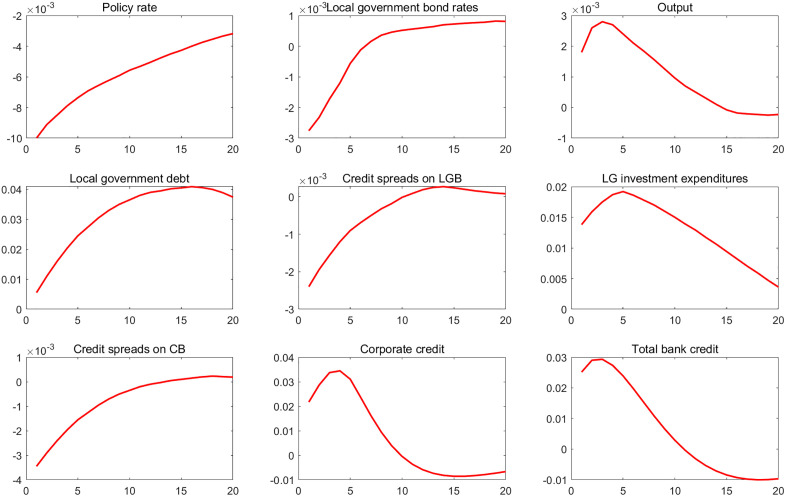
Impulse responses to monetary policy shocks: The benchmark model. The vertical axis of the graph represents numerical values, not percentages. The horizontal axis of the graph represents quarters. Interest rates and spreads are converted to annualized form. The remaining variables are in percentage deviations from steady state.

We start with an expansionary monetary policy shock, i.e., a 1 percentage point drop in the policy rate. Fig 4 shows the impulse response function of the monetary policy shock. As can be seen from the Fig 4, we have the logic of the local government debt channel of monetary policy described above: a monetary policy shock that causes the policy rate to fall causes the cost of bond issuance by local governments to fall, and local government investment spending and debt to rise, thereby stimulating output. Specifically, a 1 percentage point fall in the policy rate reduces local government borrowing costs by 0.28 percentage points and increases output by 0.18 percentage points. In addition to this, we can also see a 0.3 percentage point decline in corporate bond credit spreads, a 2.1 percentage point increase in corporate credit, and a 2.7 percentage point increase in total bank credit. It is worth noting the long-term effect of the accumulation of local government debt, as local government borrowing costs rise due to the increase in local government debt, which ultimately dampens output. The reason is that credit spreads on corporate bonds rise faster than those on local government bonds, which in turn makes banks allocate more to local government bonds and crowd out corporate credit, leading to less corporate investment and ultimately less output. In addition to the economic reasons mentioned above, institutionally, banks also prefer government bonds, which makes it easier to meet the financing needs of local government investment expenditures.

### The impact of suppressed monetary policy responses

**Fig 5:**
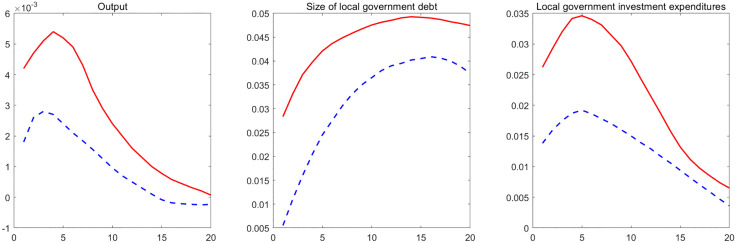
Comparison of responses to monetary policy shocks in the benchmark model (blue dashed line) versus the unrestricted model (red solid line). The vertical axis of the graph represents numerical values, not percentages. The horizontal axis of the graph represents quarters. The variables are in percentage deviations from steady state.

In the Theoretical Models section, we find that the response of local government bond yields to monetary shocks is dampened, even less than the response of corporate bond yields. This dampened response is more moderate than one would expect. Therefore, we want to know whether the stimulus effect of monetary policy is more significant assuming that local governments go ahead and issue bonds at the risk-free rate (i.e.,$\theta^{G}=\text{0}$). Fig 5 shows the impulse response of the monetary shock when the local government issues bonds at the risk-free rate. Fig 5 illustrates that the output response when bonds are issued at the risk-free rate is more than twice that of the model with realistic borrowing costs. In particular, when the policy rate drops by 1%, the output response at the risk-free rate is 0.42%, whereas the benchmark model output response is 0.18%. Local government debt rises by 2.4 percent compared to the steady state and investment spending is significantly greater when borrowing costs are more closely tied to the risk-free rate. This significant difference suggests that the ability of local governments to borrow at the risk-free rate would greatly exaggerate the stimulus effect of monetary policy on the local economy, and it also underestimates the possibility of heterogeneity in the response of individual local governments to the stimulus, and thus ignores the regional effects of monetary policy.

### The effect of heterogeneity in local government borrowing cost responses

**Fig 6:**
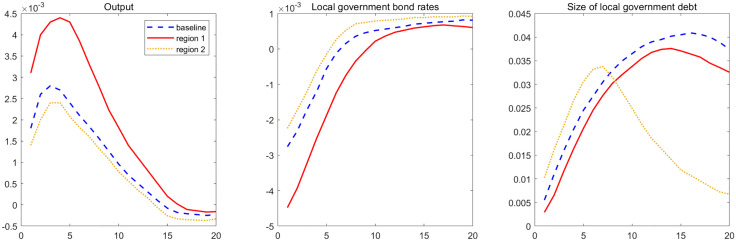
Responses to monetary policy shocks: The effect of provincial heterogeneity. The vertical axis of the graph represents numerical values, not percentages. The horizontal axis of the graph represents quarters. Interest rates and credit spreads are converted to annualized form. The remaining variables are in percentage deviations from steady state.

An important source of heterogeneity in the transmission of monetary policy among local governments is eliminated when it is assumed that the central bank's policy rate and the borrowing costs of local governments are one to one. This eliminates the possibility that the borrowing costs of local governments will react differently to monetary policy shocks. In the benchmark model, there is symmetry among local governments. To examine the impact of heterogeneity in local government borrowing cost responses, we set the borrowing cost response of region 1 to 0.43 (75th percentile) and the borrowing cost response of region 2 to 0.24, as in the benchmark model. Fig 6 illustrates the monetary shock's impulse response when there is heterogeneity in local government borrowing cost responses. As can be seen in Fig 6, the more local governments’ borrowing costs decrease following an expansionary monetary policy shock, the more output they produce and the faster their debt size increases.

**Fig 7:**
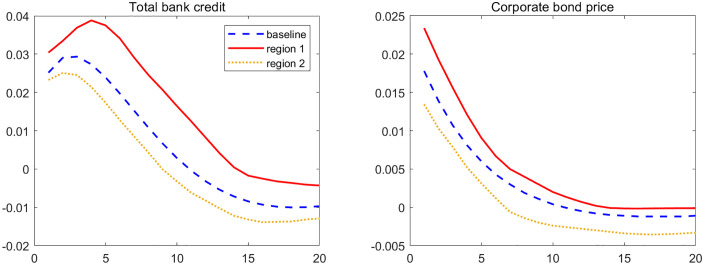
Financial spillovers across regions. The vertical axis of the graph represents numerical values, not percentages. The horizontal axis of the graph represents quarters. The variables are in percentage deviations from steady state.

It is worth noting that the borrowing cost response coefficient in the region 1 is the same as in the benchmark model, but the output growth in the region 2 is less than in the benchmark model, suggesting that there are also some cross-regional financial spillovers between local governments. Fig 7 gives some evidence that the total credit of local banks in region 1 is larger than the total credit of banks in the benchmark model and also larger than the total credit of banks in the region 2, while the total amount of credit to banks in the benchmark model is greater than the total amount of credit to banks in region 2.The price of corporate bonds in region 1 is greater than the price of corporate bonds in the benchmark model and greater than the price of corporate bonds in region 2, while at the same time the price of bonds in the benchmark model is greater than the price of corporate bonds in region 2. Financial intermediaries prefer to lend locally because bank lending in region 2 is crowded out by higher asset prices in region 1. Decreased bank credit results in lower investment, which eventually has an impact on output.

### The impact of local government competition

The “tournament” mode of fiscal expenditure competition among local governments in China is an important feature of the period of economic transition [31]. Since the reform and opening up, China's economic development model can be roughly summarized as follows: attracting foreign direct investment through competing incentives in fiscal, tax, land, and labor, promoting the full combination of foreign high-level factors of production and local low-cost factors, and then unleashing huge production capacity and the scale of exports. To a large extent, local governments that directly interfere with resource allocation directly play the role of market players. We are interested in how local government competition influences the impulse response of monetary policy shocks because it is a significant driver of economic growth but can also result in a number of issues, including duplicate construction, domestic market segmentation, and distorted resource allocation [60]. Local government competition is introduced by setting $\varphi_{y}^{*}=\text{1}$ in the model. Since the implementation of fiscal policy requires certain financial resources as a complement, local government competition will inevitably give rise to competition for debt resources, so we also introduce debt competition into the equation setting the cost of local government borrowing as a characteristic and outcome of local government competition.

**Fig 8:**
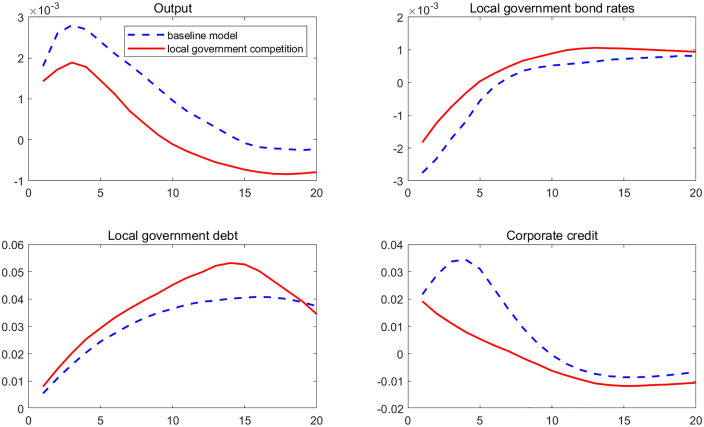
Responses to monetary policy shocks: The impact of local government competition. The vertical axis of the graph represents numerical values, not percentages. The horizontal axis of the graph represents quarters. Interest rates and credit spreads are converted to annualized form. The remaining variables are in percentage deviations from steady state.

Fig 8 shows the impulse responses of monetary policy shocks after the introduction of local government competition. As can be seen in Fig 8, compared to the baseline model, the introduction of local government competition causes output to increase less, the interest rate on local government bonds to fall less, the size of local government debt to rise more rapidly, and corporate credit to increase more slowly. It is worth noting that the introduction of local governments reduces the effectiveness of the stimulus effect of monetary shocks considerably, by roughly one-third, which is important because the excessively rapid rise in debt induced by local government competition raises the borrowing cost of local government and further crowds out business investment, which ultimately leads to less output growth. The competition among local governments reduces the stimulus effect of relaxed monetary policy. In other words, the faster drag from debt accumulation depresses output over time and shifts into negative growth space.

### Impact of real estate market adjustments

**Fig 9:**
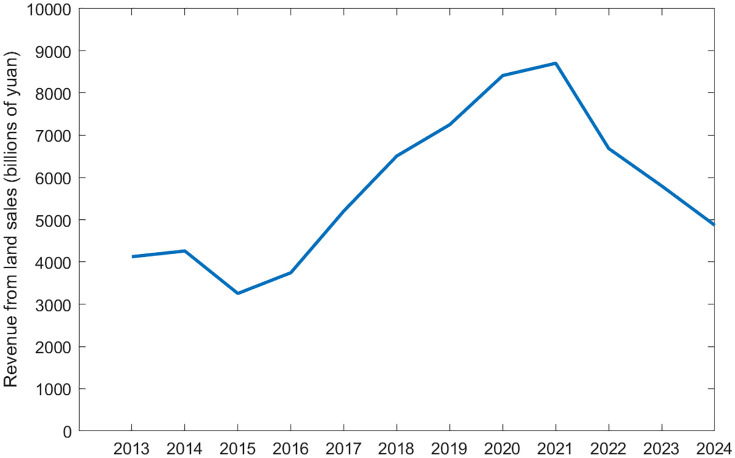
Trends in revenue from land sales.

Local governments in China are highly dependent on real estate and land-related revenues for their fiscal revenues. In recent years, due to the deep adjustment of the real estate market, there has been a significant decline in real estate and land-related revenues, which has exacerbated the conflict between the fiscal revenues and expenditures of local governments. Local government land premium revenue is the most important component of land-related revenue, accounting for about 80%. As can be seen in Fig 9, local government land premium revenues have been declining since 2021, by as much as 44%, which has compressed local government revenues and prompted local governments to rely more on the bond market for the implementation of their fiscal policies, which has further intensified the competition among local governments for financial resources and increased the financial friction faced by the local government bond market.

**Fig 10:**
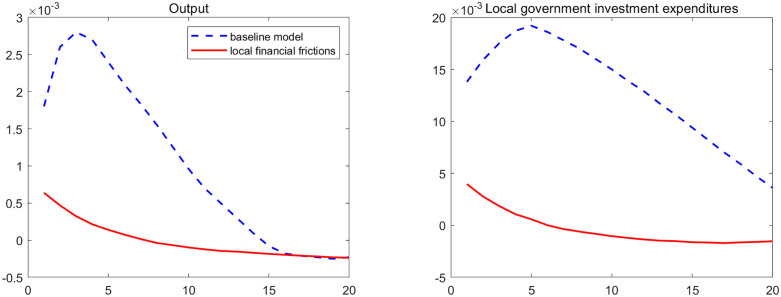
Responses to monetary policy shocks: The effect of local financial frictions. The horizontal axis of the graph represents quarters. The variables are in percentage deviations from steady state.

We can test how financial markets affect the local government bond channel of monetary policy using the model mentioned above. The escalation of financial frictions resulting from local governments’ competition for financial resources is specifically characterized as a negative shock to $\theta^{G}$, meaning that the ability of local government bond yields to decline is further inhibited, as well as an increase in $\phi^{G}$ which means that the borrowing costs of local government will become higher, which will inhibit the implementation and effectiveness of local government fiscal policy, and thus impede the transmission effect of monetary policy. Fig 10 illustrates how the transmission of monetary policy deteriorates when local governments encounter greater financial frictions. The output response is only one-third that of the baseline model, primarily because the financing constraints limit the ability of local governments to implement their fiscal policies, which in turn results in a significant decrease in their investment expenditures.

### Applicability of the model

Although the model is calibrated using Chinese data and institutional features, its underlying transmission mechanism is not unique to China. The channel through which monetary policy affects subnational borrowing costs, fiscal decisions, and macroeconomic outcomes can also operate in other fiscally decentralized economies where local governments rely on bond financing, such as the United States, Germany, Spain, Italy, Brazil, and Canada. However, some institutional assumptions embedded in the model are specific to China. In particular, the incentive structure generated by intergovernmental competition, the performance evaluation system for local officials, and the prominent role of local governments in infrastructure investment and economic development distinguish China's fiscal system from those of most other countries. These institutional features amplify local governments’ responsiveness to changes in financing costs and therefore strengthen the transmission mechanism identified in this paper.

Consequently, while the qualitative mechanism is expected to remain valid across decentralized fiscal systems, the quantitative effects should be interpreted with caution, as country-specific fiscal institutions, debt management practices, and financial market structures may alter the magnitude of the transmission mechanism. Futhermore, the model would also require recalibration of institutional parameters, such as fiscal autonomy, political incentives, and local debt management frameworks, before applying the model to other institutional contexts. Therefore, the proposed framework can be extended to other fiscal federal or fiscally decentralized economies through appropriate recalibration of country-specific institutional parameters.

Future research could extend the present framework through cross-country calibration and comparative analysis.

## Conclusions and Implications

This paper first verifies the existence of the local government bond channel of monetary policy in China through empirical analysis, and introduces the local government bond channel of monetary policy into the DSGE model and analyzes it quantitatively. Although local governments now play a bigger role in economic development due to Chinese-style fiscal and financial decentralization, the central government and local governments face varying degrees of financial friction because the central government can borrow money at a risk-free interest rate while the local government must pay a credit premium. This is due to differences in local endowments and levels of economic and financial development. This paper quantitatively analyzes this situation using a model and concludes that if local governments are able to borrow at the risk-free rate, the stimulus effect of monetary policy will be much higher, with an output response more than twice that of the baseline model. At the same time, local governments also face different levels of financial frictions and thus pay different levels of credit premiums. The quantitative results of the model show that when local governments have a yield response at the 75th percentile, their output response is more than twice as high as the average response, while the output response at the average response is lower than that of the benchmark model, which is explained by the fact that there are certain cross-regional financial spillovers between local governments, i.e., there will be a cross-regional flow of financial resources, which is one of the results of financial competition among local governments. When there is local government competition, loose monetary policy shocks cause local government bond rates to fall more slowly, debt to increase more quickly, and corporate credit to increase more slowly and turn into crowding-out effects more quickly, and ultimately output growth responses are lower.

Based on the theoretical and empirical analysis in this paper, there are three policy insights: (1) Excessive local competition has a significant impact on policy effects. Local government competition will weaken the transmission effect of monetary policy and the effect of local government fiscal policy, which is not only not conducive to dredging the transmission of monetary policy, but also not conducive to alleviating local government debt pressure. Macroeconomic control departments should pay more attention to communication and cooperation with local governments. Government departments should actively promote the establishment of a regional development mechanism for co-construction, sharing and cooperation, so that intergovernmental competition will give way to intergovernmental cooperation, accelerate the construction of a unified national market, and promote the market-oriented allocation of factor markets to improve policy efficiency. (2) When the scale of local government bonds is getting larger, the more significant the local government bond channel of monetary policy will be. When the local government borrowing cost response to monetary policy is greater, monetary policy is also more effective. There is response heterogeneity among local governments, an important reason for the regional effect of monetary policy. On the one hand, it is necessary to improve the construction of the local government bond market, reduce the financial friction in the bond market, and weaken the heterogeneity of monetary policy transmission; on the other hand, it is necessary to improve the fiscal relations between the central government and the local government, reduce the credit premium of the local government, smooth the local government bond channel of the monetary policy, and improve the policy effect. (3) Local financial frictions can constrain the effectiveness of monetary policy transmission. The central government should solve the problem of the local government fiscal gap from the perspective of long-term revenue by increasing the share of local revenue instead of relying excessively on land finance, which helps to reduce the financial friction faced by local governments due to competition for financial resources.

Future studies could cover the following topics: (1) the sustainability of local government debt and risk contagion, taking into account the effect of land-based fiscal revenues to evaluate how localized risks spread through financial and real economy channels; (2) policy coordination studies, which could introduce macroprudential tools for local government debt and investigate their coordination with monetary and fiscal policies; and (3) assessments of regional coordinated development policies, quantitatively comparing the efficacy of tools like transfer payments, financing support, and joint investment in promoting balanced development and reducing regional competition.

The following are the limitations of this study: (1) The empirical data's frequency and duration are relatively constrained. The sample size used in this study's empirical analysis is rather small because of the brief history of China's local government bond market and the statistical feature that local government fiscal statistics are mainly provided on an annual basis. This somewhat limits the ability to capture long-term dynamic impacts and the robustness of the estimation results. (2) The modeling process for the actions of the central government is still somewhat basic. Instead of endogenously modeling the central government's policy reaction function inside an explicit intertemporal optimization framework, this study takes it as exogenously given in the model specification in order to concentrate on the fundamental logic of the central-local government game. The central government's dynamic trade-off process between macroeconomic regulation and risk avoidance is not adequately captured by this simplification, despite the fact that it clarifies the incentive-compatibility mechanism for local governments.

## Supporting information

S1 Data Regression and VAR data. (XLSX)

S1 File Readme file describing the dataset. (DOCX)

## References

[1] Yao Y, Zhang MY. Performance of officials and the promotion tournament——evidence from Chinese cities. Economic Research Journal. 2013;48(01):137–50.

[2] Zhou LA. Governing China’s local officials: An analysis of promotion tournament model. Economic Research Journal. 2007;2007(07):36–50.

[3] Liu QZ, Yue MY, Li XC. Counter-cyclical adjustment of local fiscal policy: fiscal decentralization or increased issuance of local government debt. Economic Theory and Business Management. 2021;41(06):50–65.

[4] Liu R, Li N. Research on multiplier and double crowding-out effects of rising local debt intensity. Journal of Management World. 2021;37(03):51–66.

[5] Cao J, Mao J, Xu Y. Why do municipal investment bonds continue to grow? ——An empirical analysis based on new statistical scope. Finance & Trade Economics. 2019;40(05):5–22.

[6] Gilchrist S, López-Salido D, Zakrajšek E. Monetary Policy and Real Borrowing Costs at the Zero Lower Bound. American Economic Journal: Macroeconomics. 2015;7(1):77–109. doi: 10.1257/mac.20130324

[7] Sensarma R, Bhattacharyya I. Measuring monetary policy and its impact on the bond market of an emerging economy. Macroeconomics and Finance in Emerging Market Economies. 2016;9(2):109–30. doi: 10.1080/17520843.2015.1123743

[8] Burger JD, Warnock FE, Warnock VC. The effects of US monetary policy on emerging market economies’ sovereign and corporate bond markets. National Bureau of Economic Research. 2017.

[9] Szczerbowicz U. The ECB unconventional monetary policies: have they lowered market borrowing costs for banks and governments?. International Journal of Central Banking. 2018;42(42):1–30.

[10] Fang F, Si D-K, Hu D. Green bond spread effect of unconventional monetary policy: Evidence from China. Economic Analysis and Policy. 2023;80:398–413. doi: 10.1016/j.eap.2023.08.019

[11] El-Shagi M, Jiang L. Monetary policy transmission in China: dual shocks with dual bond markets. Macroecon Dynam. 2023;27(8):2229–51. doi: 10.1017/s1365100522000669

[12] Beetsma RMWJ, Jensen H. Monetary and fiscal policy interactions in a micro-founded model of a monetary union. Journal of International Economics. 2005;67(2):320–52. doi: 10.1016/j.jinteco.2005.03.001

[13] Nakamura E, Steinsson J. Fiscal Stimulus in a Monetary Union: Evidence from US Regions. American Economic Review. 2014;104(3):753–92. doi: 10.1257/aer.104.3.753

[14] Farhi E, Werning I. Fiscal Unions. American Economic Review. 2017;107(12):3788–834. doi: 10.1257/aer.20130817

[15] Wilson M. The municipal government channel of monetary policy. Macroecon Dynam. 2024;29. doi: 10.1017/s1365100524000385

[16] Carlino G, DeFina R. The Differential Regional Effects of Monetary Policy. Review of Economics and Statistics. 1998;80(4):572–87. doi: 10.1162/003465398557843

[17] Carlino G, DeFina R. The Differential Regional Effects of Monetary Policy: Evidence from the U.S. States. Journal of Regional Science. 1999;39(2):339–58. doi: 10.1111/1467-9787.00137

[18] Owyang M, Wall HJ. Structural breaks and regional disparities in the transmission of monetary policy. FRB of St. Louis. 2003.

[19] Fielding D, Shields K. Regional Asymmetries in the Impact of Monetary Policy Shocks on Prices: Evidence from US Cities*. Oxf Bull Econ Stat. 2010;73(1):79–103. doi: 10.1111/j.1468-0084.2010.00608.x

[20] Elbourne A, de Haan J. Modeling Monetary Policy Transmission in Acceding Countries: Vector Autoregression Versus Structural Vector Autoregression. Emerging Markets Finance and Trade. 2009;45(2):4–20. doi: 10.2753/ree1540-496x450201

[21] Huang JL, Qin FM. Regional asymmetries in the effects of monetary policy in China: Evidence from a mixed cross-section global vector autoregression model. Journal of Financial Research. 2017;12:1–16.

[22] Tang L, Ai G, Cai Z. Regional effects of China’s monetary policy during the economic transition period: Based on China’s city classification system under the new normal. PLoS One. 2023;18(9):e0291317. doi: 10.1371/journal.pone.0291317 37703306 PMC10499217

[23] Tsang A. Uncovering heterogeneous regional impacts of Chinese monetary policy. Empir Econ. 2024;67(3):915–40. doi: 10.1007/s00181-024-02575-2

[24] Song W, Zhong ZS. The existence and origin of regional effects of monetary policy in China——an analysis based on the theory of optimum currency areas. Economic Research Journal. 2006;(03):46–58.

[25] Guo X, Masron TA. Regional effects of monetary policy in China: evidence from China’s provinces. Bulletin of Econ Res. 2016;69(2):178–208. doi: 10.1111/boer.12095

[26] Chang HB, Xu CX. Empirical analysis of regional differences in China’s monetary policy transmission mechanism. Economic Science. 2007;2007(05):66–76.

[27] Jiang YM, Chen Z. An empirical study of the regional effects of monetary policy in the framework of the SVAR model: 1978-2006. Journal of Financial Research. 2009;2009(04):180–95.

[28] Wang XZ, Mao ZG, Liu HY. The regional effect of monetary policy: Evidence from real estate market in China. Journal of Financial Research. 2011;(09):42–53.

[29] Liao C, Wang S. The Political Economy of the Differential Regional Effects of Monetary Policy: Evidence From China. The Developing Economies. 2021;59(4):351–70. doi: 10.1111/deve.12272

[30] Chen C, Gao R, Gong LT. The regional heterogeneity effects of monetary policy transmission in China——an analysis on the origin of existing disagreement. Lanzhou Academic Journal. 2023;2023(09):58–77.

[31] Zhu J, Xu ZW. Fiscal decentralization, regional fiscal competition and macroeconomic fluctuations in China. Economic Research Journal. 2018;53(01):21–34.

[32] Wang WF, Wang ZQ, Guo LY. Fiscal decentralization and China’s unbalanced economic structure. Economic Research Journal. 2020;55(05):49–65.

[33] Xiong C, Zhou YG, Jin H. Inter-regional effects of local government implicit debt: An interbank network approach. Economic Research Journal. 2022;57(07):153–71.

[34] Mao J, Xu JW. Reality base of research on China’s local government debt——institutional transition, statistical methods and key facts. Public Finance Research. 2019;(01):3–23. doi: 10.19477/j.cnki.11-1077/f.2019.01.001

[35] Ang A, Bai J, Zhou H. The great wall of debt: Real estate, political risk, and Chinese local government financing cost. The Journal of Finance and Data Science. 2023;9:100098. doi: 10.1016/j.jfds.2023.100098

[36] Gertler M, Karadi P. Monetary Policy Surprises, Credit Costs, and Economic Activity. American Economic Journal: Macroeconomics. 2015;7(1):44–76. doi: 10.1257/mac.20130329

[37] Chen ZZ, Li L, Yu CH. Transmission of monetary policy shocks and signaling channels in China——Based on high frequency data in financial markets. China Economic Quarterly. 2023;23(01):56–73.

[38] Kamber G, Mohanty MS. Do interest rates play a major role in monetary policy transmission in China? Bank for International Settlements. 2018.

[39] Shen XB, Chen Y, Lin BQ. The impacts of technological progress and industrial structure distortion on China’s energy intensity. Economic Research Journal. 2021;56(02):157–73.

[40] Miao XL, Wang T, Gao YG. The effect of fiscal transfer on the gap between urban-rural public services based on a grouping comparison of different economic catching-up provinces. Economic Research Journal. 2017;52(02):52–66.

[41] Wang KL, Zhao B, Xu RY. Local government competition and manufacturing value chain climbing. Eastern Forum-Journal of Qingdao University (Social Science Edition). 2023;(06):105–19.

[42] Adelino M, Cunha I, Ferreira MA. The Economic Effects of Public Financing: Evidence from Municipal Bond Ratings Recalibration. The Review of Financial Studies. 2017;30(9):3223–68. doi: 10.1093/rfs/hhx049

[43] Ramey VA. Identifying Government Spending Shocks: It’s all in the Timing*. The Quarterly Journal of Economics. 2011;126(1):1–50. doi: 10.1093/qje/qjq008

[44] Sims E, Wu JC. Evaluating Central Banks’ tool kit: Past, present, and future. Journal of Monetary Economics. 2021;118:135–60. doi: 10.1016/j.jmoneco.2020.03.018

[45] Gertler M, Karadi P. A model of unconventional monetary policy. Journal of Monetary Economics. 2011;58(1):17–34. doi: 10.1016/j.jmoneco.2010.10.004

[46] Bi H, Traum N. Unconventional monetary policy and local fiscal policy. European Economic Review. 2023;156:104476. doi: 10.1016/j.euroecorev.2023.104476

[47] Krenz J. Unconventional monetary policy in a monetary union. In: Discussion Paper, 2022.

[48] Alpanda S, Kabaca S. International Spillovers of Large-Scale Asset Purchases. Journal of the European Economic Association. 2019;18(1):342–91. doi: 10.1093/jeea/jvy053

[49] Schmitt-Grohé S, Uribe M. Closing small open economy models. Journal of International Economics. 2003;61(1):163–85. doi: 10.1016/s0022-1996(02)00056-9

[50] Ai F, Wang WF. Tax sharing, regional competition and local government debt expansion. Modern Economic Science. 2021;43(06):1–14.

[51] Bodenstein M, Guerrieri L, LaBriola J. Macroeconomic policy games. Journal of Monetary Economics. 2019;101:64–81. doi: 10.1016/j.jmoneco.2018.07.015

[52] Mei DZ, Cui XY, Wu Y. House price fluctuation, land finance and business cycle in China. Economic Research Journal. 2018;53(01):35–49.

[53] Lv CF, Huang MB. Habit formation, borrowing constraints and the real business cycle in China: An empirical study on a RBC model. Journal of Financial Research. 2011;(09):1–13.

[54] Liu K. How the land system with Chinese characteristics affects China’s economic growth – an analysis based on a multisector dynamic general equilibrium framework. CPE. 2020;3(1):225–54. doi: 10.1108/cpe-05-2020-0009

[55] Hao DP, Wang B, Li L. Fed policy changes, international capital flows, and macroeconomic fluctuations. Journal of Financial Research. 2020;2020(07):38–56.

[56] Shi SB, Yin ZD, Tang YG. Fiscal decentralization, financial constraints and tax policy cyclicality. Economic Research Journal. 2019;54(09):90–105.

[57] Lv BY, Ma GR, Hu S. The lion’s share: A key indicator of China’s fiscal decentralization. Finance & Trade Economics. 2021;42(08):20–36.

[58] Poutineau J-C, Vermandel G. Cross-border banking flows spillovers in the Eurozone: Evidence from an estimated DSGE model. Journal of Economic Dynamics and Control. 2015;51:378–403. doi: 10.1016/j.jedc.2014.11.006

[59] He Z, Krishnamurthy A. A Macroeconomic Framework for Quantifying Systemic Risk. American Economic Journal: Macroeconomics. 2019;11(4):1–37. doi: 10.1257/mac.20180011

[60] Lu M, Chen Z, Yan J. Increasing return, development strategy and regional economic segmentation. Economic Research Journal. 2004;2004(01):54–63.

