# Supplementary material for: The local government bond channel of China's monetary policy
Source: PLoS One. 2026 Aug 18;21(8):e0354727. doi: 10.1371/journal.pone.0354727 (PMC13485021; doi:10.1371/journal.pone.0354727)
Supplement: Supplementary file 1 [file pone.0354727.s002.docx]

Sheet1 for figure1

Sheet2 for figure2

Sheet3 for table1 and table2

Sheet4 is monetary policy shock, which is used for other regressions.

Sheet8 for table3

Sheet5 for table4

Sheet6 for table5

Sheet7 for Proxy VAR
